# K_Ca_3.1 K^+^ Channel Expression and Function in Human Bronchial Epithelial Cells

**DOI:** 10.1371/journal.pone.0145259

**Published:** 2015-12-21

**Authors:** Greer K. Arthur, S. Mark Duffy, Katy M. Roach, Rob A. Hirst, Aarti Shikotra, Erol A. Gaillard, Peter Bradding

**Affiliations:** Department of Infection, Immunity and Inflammation, Institute for Lung Health, University of Leicester, Leicester, United Kingdom; University of Alabama at Birmingham, UNITED STATES

## Abstract

The K_Ca_3.1 K^+^ channel has been proposed as a novel target for pulmonary diseases such as asthma and pulmonary fibrosis. It is expressed in epithelia but its expression and function in primary human bronchial epithelial cells (HBECs) has not been described. Due to its proposed roles in the regulation of cell proliferation, migration, and epithelial fluid secretion, inhibiting this channel might have either beneficial or adverse effects on HBEC function. The aim of this study was to assess whether primary HBECs express the K_Ca_3.1 channel and its role in HBEC function. Primary HBECs from the airways of healthy and asthmatic subjects, SV-transformed BEAS-2B cells and the neoplastic H292 epithelial cell line were studied. Primary HBECs, BEAS-2B and H292 cells expressed K_Ca_3.1 mRNA and protein, and robust K_Ca_3.1 ion currents. K_Ca_3.1 protein expression was increased in asthmatic compared to healthy airway epithelium in situ, and K_Ca_3.1 currents were larger in asthmatic compared to healthy HBECs cultured *in vitro*. Selective K_Ca_3.1 blockers (TRAM-34, ICA-17043) had no effect on epithelial cell proliferation, wound closure, ciliary beat frequency, or mucus secretion. However, several features of TGFβ1-dependent epithelial-mesenchymal transition (EMT) were inhibited by K_Ca_3.1 blockade. Treatment with K_Ca_3.1 blockers is likely to be safe with respect to airway epithelial biology, and may potentially inhibit airway remodelling through the inhibition of EMT.

## Introduction

Asthma is a common disease affecting 5–10% of Westernised populations, and an important cause of morbidity and mortality at all ages [1]. For approximately 10% of patients with asthma, current therapies are of poor efficacy: in consequence novel approaches to treatment are urgently required. Ion channels are emerging as interesting therapeutic targets in both inflammatory and structural non-excitable cells. Channels carrying K^+^, Cl^-^, and Ca^2+^ mediate a variety of cell processes including proliferation [2], differentiation [3], adhesion [4], mediator release [5] and migration [6]. The Ca^2+^-activated K^+^ channel K_Ca_3.1 is of particular interest as a novel target for asthma therapy [7–9].

The airway epithelium is at the interface between the airway and the external environment, and is the first structure to interact with noxious stimuli such as allergens, viruses and pollutants. Not only does the columnar epithelium tend to shed from the basal layer, the airway epithelium is functionally abnormal in asthma [10–12]. Epithelial repair normally involves upregulation of the epithelial growth factor (EGF) receptor which drives the repair response. Interestingly in asthmatic epithelium, the proliferative repair response is impeded, but other consequences of EGF receptor activation remain intact. Thus there is on-going release of pro-inflammatory cytokines which may promote cellular recruitment, and there is release of profibrogenic growth factors which may drive the remodelling response [10–12].

K_Ca_3.1 channels are reported to be expressed by several epithelia including primary human renal epithelial cells [13], and human bronchial epithelial cell (HBEC) cell lines (Calu-3, Nu-Li, Cu-Fi) [14–17]. Pharmacological studies have also suggested it is expressed in primary HBECs, but these were not definitive [18]. The proposed role for K_Ca_3.1 in Calu-3 cells is to reduce HCO^3-^ secretion and increase Cl^-^ secretion, and hence promote airway surface liquid hydration [14]. It has therefore been suggested that K_Ca_3.1 openers might be useful in the treatment of cystic fibrosis. K_Ca_3.1 has also been implicated in several cell processes including proliferation [19,20], migration [6] and regulation of cytokine production [21], effects mediated through its control of cell membrane potential which in turn regulates Ca^2+^ signals [21,22]. If K_Ca_3.1 is expressed in primary HBEC, it is therefore unclear whether its inhibition might be deleterious in asthma through the inhibition of epithelial cell wound healing, proliferation, airway lining fluid secretion and ciliary beat, or beneficial though the inhibition of chemokine/cytokine production, mucus secretion and epithelial mesenchymal transition (EMT).

We have investigated K_Ca_3.1 channel expression in HBEC cell lines and primary HBECs obtained from bronchoscopic bronchial brushings from both healthy and asthmatic subjects, and in bronchial biopsies. Functional responses in primary HBECs have been examined using the specific K_Ca_3.1 blockers TRAM-34 [19] and ICA-17043 (Senicapoc) [23].

## Materials and Methods

### Subjects

Healthy control subjects and subjects with asthma were recruited from respiratory clinics and hospital staff and by means of local advertising. Asthma was defined using standard criteria as described previously [24]. Asthma severity was defined by British Guideline on the Management of Asthma treatment steps (mild = step 1, β_2_-agonist only; moderate = steps 2 and 3, inhaled corticosteroid ≤800 mg beclomethasone equivalent per day ± long-acting β_2_-agonist; severe = step 4 and 5) [25]. Healthy subjects had no history of respiratory disease. All subjects undergoing bronchoscopy were nonsmokers with a past smoking history of less than 10 pack-years and were free from asthma exacerbations for at least 6 weeks. The study was approved by the National Research Ethics Committee East Midlands-Northampton (reference 04/Q2502/74) and Leicestershire, Northamptonshire and Rutland (08/H0406/189). All patients provided written informed consent.

### Primary epithelial cell isolation and culture

HBECs were isolated from bronchial brushings obtained at bronchoscopy, and cultured using methodology adapted from that described previously [26]. Briefly, brush samples obtained from bronchoscopies were grown on collagen type I-coated (30 μg/ml, PureCol; Advanced Biomatrix, Carlsbad, CA, USA) 12-well tissue culture trays in bronchial epithelial growth media (BEGM; Lonza, Basel, Switzerland) supplemented with a SingleQuot kit (Lonza) and antibiotic-antimycotic solution (Invitrogen, Paisley, UK) until confluent. The confluent un-ciliated basal cells were expanded into PureCol-coated 75 cm^2^ flasks and the BEGM was replaced every 2–3 days. Basal cells were characterised for expression of cytokeratin 5 and 14 (Ab16570; Abcam, Cambridge, UK) using immunofluorescence. To induce HBEC differentiation, basal cells were seeded at approximately 2 x 10^5^ cells per cm^2^ onto the apical chamber of PureCol-coated polyester Transwells (12 mm diameter, 0.4 μm-pore size, 12-well plate, Transwell Clear; Corning Costar, Tewksbury, MA, USA) in BEGM until confluent. Upon reaching confluence, the basal cell monolayer was fed on the basolateral side only with air liquid interface (ALI) media: 50% BEGM and 50% Hi-glucose minimal essential medium (Invitrogen) containing 100 nM retinoic acid (Sigma, Dorset, UK). The media was exchanged every 2–3 days and the apical surface secretions were gently removed by gentle washing with phosphate buffered saline (PBS; Invitrogen). Cilia were observed after a minimum of 21 days, and analysed through high-speed video microscopy.

### H292 cells

The human mucoepidermoid lung cancer cell line NCI-H292 (herein referred to as H292) was purchased from the American Type Culture Collection (ATCC, Manassas, VA, USA). H292 cells were cultured in RPMI 1640 media (LGC Standards, Middlesex, UK) supplemented with 10% fetal bovine serum (Invitrogen) and antibiotic-antimycotic solution (Invitrogen) in a 5% CO_2_ incubator. Cultures were sub-cultured every 7 days by treatment with trypsin-EDTA solution (Sigma). For establishment of ALI, H292 cells were plated (2 x 10^5^ cells per well) on non-coated polyester Transwell membranes (12 mm diameter, 0.4 μm-pore size, 12-well plate, Transwell Clear; Corning Costar) and maintained in RPMI 1640 until confluent. Once confluent, media from the apical chamber of the Transwell was removed and only applied to the basolateral chamber to establish the ALI. Cells were maintained at ALI for 48 h before being used for experiments.

### BEAS-2B cells

The human virus-transformed bronchial epithelial cell line BEAS-2B was purchased from ATCC and cultured in BEGM media (Lonza), supplemented with a SingleQuot kit (Lonza) and antibiotic-antimycotic solution (Invitrogen) in a 5% CO_2_ incubator. All culture containers were pre-coated with 30 μg/ml PureCol (Advanced BioMatrix).

### Ciliary beat frequency, ciliary immotility index and ciliary dyskinesia measurements in primary HBECs

The differentiated primary HBEC cultures were removed from the insert by gentle scraping with a spatula, as described previously [26]. The ciliated cells were then incubated in a suspension of M199 HEPES containing DMSO (vehicle control) or TRAM-34 (200 nM) for 30 and 60 minutes at 37°C. For cilia readings, 100 μl of cell suspension was placed into a chamber slide and cilia were viewed and recorded at 1000x magnification at 37°C. Measurements were made as described previously [26,27]. Briefly, beating cilia were recorded (1000x magnification) using a MotionPro x4 high speed video camera (Lake Image Systems Ltd, Tring, UK) at 500 frames/sec. Video sequences were played back either at reduced frame rates or frame by frame and ciliary beat frequency was determined by timing a pre-selected number of individual ciliary beat cycles. Slow motion playback of the high-speed video recordings also enabled us to determine the number of immotile cilia (% of epithelial strip) and the number of dyskinetic cilia (% of epithelial strip).

### Quantitative Real Time PCR

Total RNA isolated from cell cultures using the RNeasy kit (Qiagen, Manchester, UK) and first strand cDNA synthesis was conducted using the RevertAid kit (Fisher Scientific, Loughborough, UK), as per the manufacturer’s instructions. Quantitative real time PCR (qPCR) was performed to assess the quantity of mRNA encoding K_Ca_3.1 or MUC5AC using first strand cDNA samples produced from primary HBEC and H292 cell total RNA. Detection of K_Ca_3.1 mRNA was performed using the Fast SYBR Green Master Mix (Applied Biosystems, Paisley, UK) with K_Ca_3.1 (Hs_KCNN4_1_SG Quantitect Primer Assay, QT00003780; Qiagen), alongside primers targeting the internal normaliser gene β-actin (Hs_ACTB_1_SG Quantitect Primer Assay, QT00095431; Qiagen). Detection of MUC5AC mRNA was performed using Gene Expression Master Mix (Applied Biosystems) and TaqMan probes targeting MUC5AC mRNA (Hs00873651_mH, catalogue number 4331182; Applied Biosystems), alongside TaqMan probes targeting the internal normaliser gene ribosomal 18S (Hs99999901_s1, catalogue number 4331182; Applied Biosystems). All expression data were corrected using the reference dye ROX and normalised to β-actin and ribosomal 18S mRNA, as required, using the ΔC_T_ method: ΔC_T_ = C_T_(gene of interest)–C_T_(internal control). For relative quantification to assess the fold change caused by different treatment conditions, the ΔΔC_T_ method was used: ratio = 2^-[(C_T_ (gene of interest)-C_T_ (internal control)] (2^−ΔΔCT^). All reactions were run in triplicate on a Stratagene Mx3000P real-time thermocycler (Agilent Technologies, Santa Clara, CA, USA).

### Western blotting

K_Ca_3.1 protein expression in primary HBECs was analysed by Western blotting as described previously [28]. Soluble protein from equivalent numbers of cells was resolved by 10% SDS-PAGE and then transferred to a nitrocellulose membrane. Membranes were blocked and assayed by immunoblot using 1 μg/ml anti-K_Ca_3.1 antibody (Sigma). Protein bands were identified by goat anti-rabbit IgG horseradish peroxidase (HRP)-linked secondary antibody (Dako, Ely, UK). Bands were visualised by chemiluminescence with Pierce ECL substrate (Invitrogen) and the ImageQuant LA S 4000 (GE Healthcare Life Sciences, Little Chalfont, UK). Β-actin was used as a positive control for the western blot. To re-stain blots, bound primary and secondary antibodies were removed by incubating the membrane in 0.2 M NaOH for 5 min. Membranes were washed, blocked, probed with 6.7 ng/ml primary monoclonal β-actin antibody HRP conjugate (Santa Cruz Biotechnology, Dallas, Texas, USA) for 2 h at room temperature, and then visualised by chemiluminescence as described above.

### Immunohistochemistry

Healthy control subjects and patients with asthma underwent fiberoptic bronchoscopy with bronchial biopsy [29]. Biopsies were fixed in phenyl methyl sulphonyl fluoride (PMSF), iodoacetamide and acetone at -20°C and then embedded in glycol methacrylate (GMA), as described previously [30]. GMA sections (2 μm thickness) were immunostained for K_Ca_3.1 (rabbit polyclonal IgG anti-human K_Ca_3.1, 5 μg/ml; Sigma) and MUC5AC (mouse monoclonal IgG1 anti-human MUC5AC, 20 μg/ml; Merck Millipore, Billerica, MA, USA), as described previously [30]. Isotype controls (rabbit IgG; BD Biosciences, Oxford, UK; mouse IgG1; Dako) were also tested at the corresponding concentrations. Immunohistochemical analysis of paraffin-embedded H292 ALI cultures was also undertaken using the same antibodies and controls described above for MUC5AC. Sections were counter-stained using Mayer’s haematoxylin and visualised using a light microscope (Olympus, Southend-on-Sea, UK).

Images were collected and the epithelial areas in biopsy sections were identified and measured using Cell^F^ software version 5.0 (Olympus). Cells stained in sequential sections of bronchial biopsy specimens were co-localised using computer analysis. The quantity of positive staining in epithelial areas of each biopsy and in H292 ALI culture sections were assessed using a thresholding technique [29], based on the hue, saturation and intensity of K_Ca_3.1 or MUC5AC staining.

### Patch clamp electrophysiology

The whole-cell variant of the patch-clamp technique was used [5,31]. Patch pipettes were made from borosilicate fiber-containing glass (Clark Electromedical Instruments, Edenbridge, UK), and their tips were heat polished, typically resulting in resistances of 6–8 MΩ. The standard pipette solution contained (in mM) KCl, 140; MgCl_2_, 2; HEPES, 10; Na^+^-ATP, 2; GTP, 0.1 (pH 7.3). The standard external solution contained (in mM) NaCl, 140; KCl, 5, CaCl_2_, 2; MgCl_2_, 1; HEPES, 10 (pH 7.3). For recording, HBECs, H292 or BEAS-2B cells were detached using trypsin-EDTA solution, centrifuged, and resuspended in fresh media. Aliquots of the cell suspension were placed in 35 mm dishes containing standard external solution. Whole-cell currents were recorded using an Axoclamp 200A amplifier (Axon Instruments, Sunnyvale, CA, USA), and currents were evoked by applying voltage commands to a range of potentials (-120 to 100 mV) in 10 mV steps from a holding potential of -20 mV. Currents were digitized (sampled at a frequency of 10 kHz), stored on computer, and subsequently analysed using pClamp software (version 10.3; Molecular Devices, Sunnyvale, CA, USA). Capacitance transients were minimized using the capacitance neutralization circuits on the amplifier. Correction for series resistance was not routinely applied. Experiments were performed with a perfusion system (Automate Scientific, San Francisco, USA) to allow solution changes, although drugs were added directly to the recording chamber.

### BEAS-2B and H292 cell proliferation

Cell proliferation assays were performed on H292 cells using the CyQUANT cell proliferation assay kit (Invitrogen) and on BEAS-2B cells using the MTS assay (Promega, Southampton, UK). For all experiments, cells were treated with K_Ca_3.1 blockers (TRAM-34, 200 nM; ICA-17043, 100 nM) or 0.1% DMSO (vehicle control) for 24–72 h. Assays were performed alongside a cell number standard curve consisting of known numbers of cells. Each experimental condition was run in triplicate and each experiment repeated three times.

### Epithelial cell wound healing

Confluent HBEC monolayers in 6-well plates were scratched as described previously using a p200 pipette tip [32]. Three wounds were scratched in each monolayer, creating a linear cell-free area. 24 h prior to wounding, cells were maintained in BEGM without EGF, epinephrine or hydrocortisone due to the ability of these molecules to regulate wound repair. Cells were treated with TRAM-34 at 20 nM, 200 nM and 2 μM alongside 0.1% DMSO (vehicle control) for 24 h. Cells proliferating and migrating into the wound were observed in photographs taken over 24 h. Healing was quantified by blinding the photographs and measuring the area of the wound using Cell^F^ software and reported as a percentage of the starting area of the wound. Each experiment was repeated four times.

### Epithelial mucus production and secretion

To assess mucin production and secretion, H292 cells were cultured at ALI. To analyse intracellular mucin content, lysates of H292 ALI cultures were collected by freezing cultures in 350 μl pure water at -80°C. To analyse the quantity of secreted mucins, apical washes of H292 ALI cultures were collected by incubating the surface of the ALI cultures with 350 μl warm sterile PBS, washing the PBS over the cultures by pipetting, and then storing at -80°C.

Mucin content of H292 cell lysates and apical washes was assessed semi-quantitatively using an enzyme-linked lectin assay (ELLA), as described previously [33,34]]. To detect the presence of mucin glycoproteins, a biotinylated lectin from the snail Helix pomatia (Sigma) which recognises terminal alpha *N*-acetylgalactosamine (GalNAc) residues was used [33,35,36]. Briefly, 100 μl of lysate or apical wash sample was incubated with 100 μl carbonate-bicarbonate buffer (1 capsule dissolved in 100 ml pure water; Sigma) in a sealed Maxisorp plate (Nunc; Thermo Fisher Scientific) for 2 h at 37°C. The plate was washed 3 times with 0.05% Tween20 (Sigma) in Ca^2+^- and Mg^2+^-free Dulbecco’s PBS (D-PBS; Sigma). Plates were blocked for 1 h at 37°C with 100 μl blocking buffer (1% w/v gelatine in D-PBS/0.05% Tween20) in a sealed plate. The plate was washed 3 times and incubated for 1 h at 37°C with 50 μg/ml lectin. Mucin-bound lectin was detected by incubation with 100 μl streptavidin-HRP solution (R&D Systems, Abingdon, UK) for 20 min at room temperature on a rocker, and colour was developed with 3,3’,5,5’-tetramethylbenzidine (TMB substrate; BD Biosciences). The reaction was stopped with 2 N H_2_SO_4_ and absorbance was read at 450 nm using an EnSpire plate reader (Perkin Elmer, Coventry, UK). Mucin from bovine submaxillary glands (Sigma) was used as a standard and positive control.

### Epithelial-mesenchymal transition (EMT)

EMT experiments were performed on BEAS-2B cells using immunofluorescence. BEAS-2B cells were cultured on Lab-Tek plastic chamber slides (Thermo Fisher Scientific) pre-coated with 30 μg/ml PureCol (Advanced BioMatrix). Cells were grown to approximately 80% confluence before experimental conditions were applied. Cells were maintained for 30 min with K_Ca_3.1 blockers (TRAM-34, 200 nM; ICA-17043, 100 nM) or 0.1% DMSO (vehicle control) before being treated with 10 ng/ml TGFβ1 (R&D Systems) in the presence of fresh K_Ca_3.1 blockers or 0.1% DMSO. After 72 h, cells were fixed with ice-cold methanol (Sigma) for 20 min on ice and then left to air-dry at room temperature. Chamber slides were blocked with PBS-3% BSA and then stained with primary antibodies: 5 μg/ml mouse monoclonal IgG2b anti-E-cadherin antibody clone 180224 (R&D Systems); 5 μg/ml monoclonal mouse anti-vimentin IgG1 antibody (Sigma); or 5 μg/ml rabbit polyclonal IgG anti-collagen type 1 antibody (Merck Millipore). Successful binding of primary antibodies was identified using secondary antibodies: 88 μg/ml rabbit anti-mouse antibody conjugated with FITC (Dako); or 50 μg/ml goat anti-rabbit antibody conjugated with FITC (Sigma). Cells were counterstained with 4’,6-diamidino-2-phenylindole (DAPI). As a negative control, slides were stained with isotype control antibodies: mouse IgG2b (R&D Systems), mouse IgG1 (Dako) or rabbit IgG (BD Biosciences), at corresponding concentrations. Images were captured on an epifluorescent microscope (Olympus BX50) and analysed using ZEN imaging software (Zeiss, Cambridge, UK). Matched exposures were used for isotype controls.

To assess shape change, vimentin-stained cells were analysed blind by measuring the length/width ratio. The intensity of E-cadherin and collagen-1 staining were quantified as gray value (green fluorescence extracted) using ZEN imaging software (Zeiss). For all antibodies, six areas per condition were analysed, and each experiment was repeated at least three times on separate occasions.

### Statistical analysis

Data across groups were compared with either the ANOVA or Kruskall Wallis tests where appropriate. Between group comparisons were analysed using Students unpaired/paired *t* test, or Mann Whitney U/Wilcoxon Signed Rank for paired and unpaired parametric and non-parametric data, respectively. Data were analysed with GraphPad Prism 6 (GraphPad Software, Inc., La Jolla, CA, USA). P < 0.05 was taken as statistically significant.

## Results

### Human bronchial epithelial cells express K_Ca_3.1 mRNA and protein

**Fig 1:**
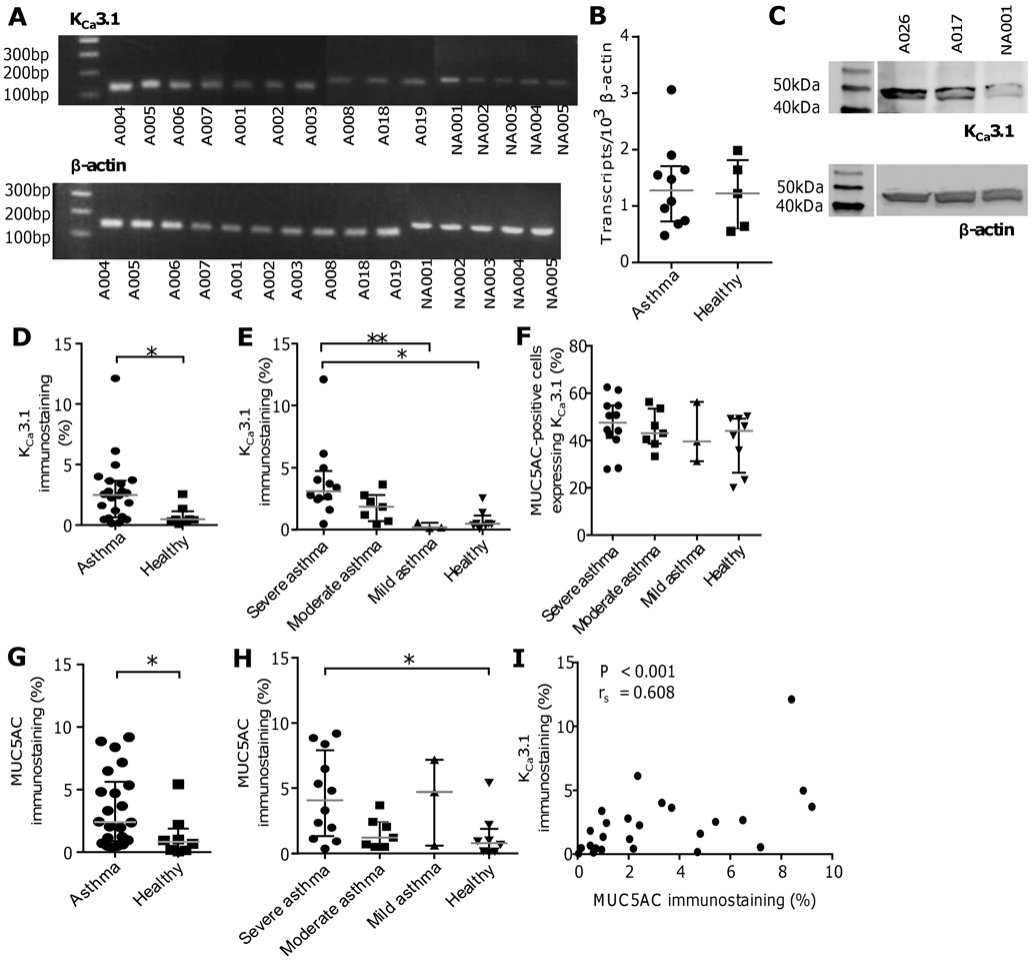
Human bronchial epithelial cells express K_Ca_3.1 mRNA and protein, and K_Ca_3.1 expression is upregulated in the asthmatic bronchial epithelium. **(A)** K_Ca_3.1 mRNA (predicted PCR product size: 130 bp) was detected in monolayers of primary HBECs isolated from both patients with asthma (n = 10; denoted with “A”) and non-asthmatic healthy controls (n = 5; denoted with “NA”), alongside the housekeeping gene β-actin (predicted PCR product size: 146 bp). (**B**) qPCR revealed that K_Ca_3.1 mRNA was expressed at similar levels in primary HBECs isolated from patients with asthma (n = 10) and healthy controls (n = 5). (**C**) An immunoreactive band of the appropriate size (K_Ca_3.1: 48 kDa; β-actin: 42 kDa) was detected in lysates of primary HBECs isolated from two patients with asthma and one healthy control. (**D**) Quantification of K_Ca_3.1 immunostaining by threshold analysis revealed that K_Ca_3.1 expression was significantly elevated in asthmatic bronchial epithelium (*P = 0.007). (**E**) K_Ca_3.1 immunostaining was significantly increased in severe asthma compared to mild asthma (**P = 0.008) and compared with healthy controls (*P = 0.002). (**F**) K_Ca_3.1 and MUC5AC immunostaining co-localised in bronchial epithelial cells of sequential sections of biopsies from patients with asthma and healthy controls. (**G**) Quantification of MUC5AC immunostaining by threshold analysis revealed that MUC5AC expression was significantly increased in asthmatic bronchial epithelium (*P = 0.030), and (**H**) this was driven by a significant difference between the severe asthma and healthy control groups (*P = 0.034). (**I**) A significant correlation was found between K_Ca_3.1 and MUC5AC immunostaining across the different severities of asthma and the healthy control groups (P < 0.001; r_s_ = 0.608). All data are plotted as median ± interquartile range; horizontal bars represent medians.

qPCR was performed on primary HBEC cell monolayers. All cultures assessed by qPCR (n = 5 healthy controls, n = 10 asthmatic donors) demonstrated expression of K_Ca_3.1 mRNA (**Fig 1A**). K_Ca_3.1 mRNA expression levels were similar between asthma and health (**Fig 1B**, data in **S1 Table**), and K_Ca_3.1 mRNA was also readily detectable in the H292 and BEAS-2B cell lines (not shown). K_Ca_3.1 protein expression by HBECs (n = 3; **Fig 1C**, data in **S1 Fig**) and H292 and BEAS-2B cells (data not shown) was demonstrated in Western blots. HBEC, H292 and BEAS-2B lysates contained an immunoreactive protein of approximately 48 kDa, the predicted size of K_Ca_3.1 [37]].

K_Ca_3.1 protein was expressed strongly in the airway epithelium in bronchial biopsies obtained from both healthy (n = 12), and mild (n = 3), moderate (n = 7), and severe (n = 8) asthmatic subjects (**Fig A in S2 Fig**). Isotype controls were negative (**Fig A in S2 Fig**). Overall K_Ca_3.1 immunostaining was increased in the epithelium of patients with asthma (P = 0.007; **Fig 1D**, data in **S2 Table**), driven largely by an increase in the severe group (P = 0.002 for severe asthma compared to healthy controls; P = 0.008 compared to mild asthma; **Fig 1E**, data in **S3 Table**). Colocalisation with MUC5AC in sequential sections demonstrated that K_Ca_3.1 immunostaining was evident in both columnar epithelial cells and goblet cells (**Fig 1F**, data in **S4 Table**). MUC5AC immunostaining was also increased in asthma compared to health (P = 0.030; **Fig 1G**, data in **S5 Table** and **Fig B in S2 Fig**), again driven by severe asthma (P = 0.034 for severe asthma compared to healthy controls; **Fig 1H**, data in **S6 Table**). Immunostaining for MUC5AC and K_Ca_3.1 were strongly correlated (r_s_ = 0.608, P < 0.001; **Fig 1I**, data in **S7 Table**).

### Human airway epithelial cells express functional K_Ca_3.1 channels

To elicit K_Ca_3.1 currents we used the K_Ca_3.1 opener 1-ethyl-2-benzimidazolinone (1-EBIO; 100 μM) (Tocris, Avonmouth, UK), as described previously in human lung mast cells [22,38], fibrocytes [39], fibroblasts [40], and airway smooth muscle cells [20]. This compound opens K_Ca_3.1 with a half-maximal value of approximately 30 μM for heterologously expressed K_Ca_3.1, with a maximal effect at about 300 μM [41].

#### Monolayers of primary HBECs

**Fig 2:**
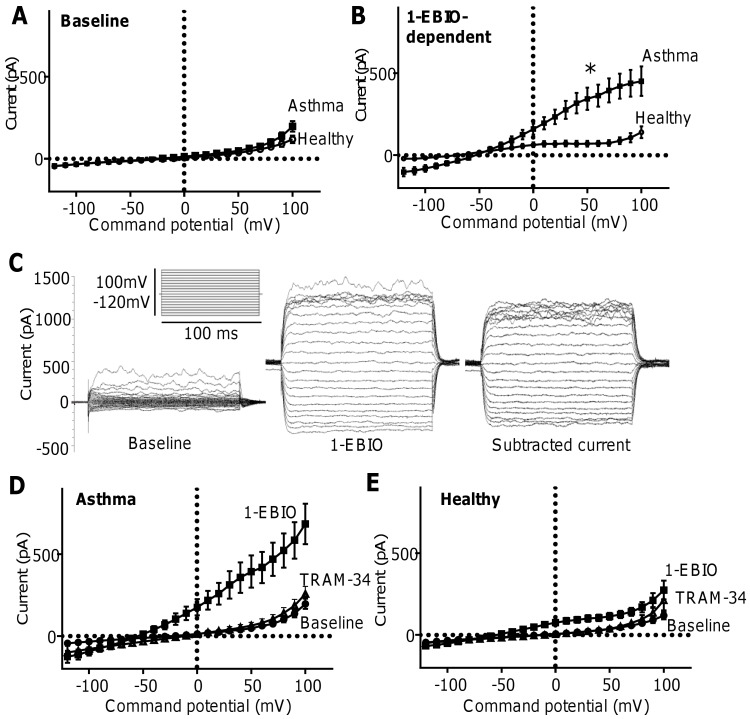
Asthmatic HBECs exhibit significantly larger K_Ca_3.1 currents than healthy HBECs. (**A**) Current-voltage curves demonstrating that baseline whole cell membrane currents recorded from asthmatic and healthy primary HBECs were similar. (**B**) The 1-EBIO-dependent (1-EBIO minus baseline) currents recorded from asthmatic HBECs (n = 29 cells from 8 donors) were significantly larger than those from healthy controls (n = 16 cells from 5 donors); *P = 0.006 at +40 mV. (**C**) Raw 1-EBIO-dependent current trace demonstrating characteristic features of K_Ca_3.1. The voltage protocol is shown inset. The K_Ca_3.1 channel blocker TRAM-34 (200 nM) inhibited currents induced by 1-EBIO in both (**D**) asthmatic HBECs (n = 19 cells from 8 donors) and (**F**) healthy HBECs (n = 14 cells from 5 donors). Data are presented as mean ± SEM.

At baseline, both asthmatic and healthy primary HBECs displayed similar membrane currents with slight outward rectification at positive potentials (Fig 2A, data in S8 Table). Outward currents at +40 mV (asthmatic HBECs: 39.7 ± 11.6 pA; healthy HBECs: 27.4 ± 5.4 pA) and reversal potentials (asthmatic HBECs: -20.0 ± 5.0 mV; healthy HBECs: -18.8 ± 7.4 mV) were not significantly different.

Similar percentages of asthmatic HBECs (37.5 (0–60.0) % of 79 cells studied from 8 donors) and healthy HBECs (52.3 (11.1–66.7) % of 35 cells studied from 5 donors) responded to 1-EBIO with the development of characteristic K_Ca_3.1 whole cell currents. The 1-EBIO-dependent currents (1-EBIO minus baseline) recorded at +40 mV were significantly larger (P = 0.006) in asthmatic HBECs (298.8 ± 60.1 pA; n = 29 cells) compared to healthy HBECs (70.1 ± 17.1 pA; n = 16 cells) (**Fig 2B**, data in **S9 Table**). The reversal potentials of the 1-EBIO-dependent currents were similar in asthmatic and healthy HBECs (-56.2 ± 3.3 mV and -63.7 ± 4.3 mV, respectively). The 1-EBIO-induced currents demonstrated the classical electrophysiological features of K_Ca_3.1 in that it appeared immediately as voltage steps were applied, did not decay during a 100 msec pulse, and demonstrated inward rectification from membrane potentials positive to about +40 mV (**Fig 2C**). Furthermore, the currents were completely blocked by the selective K_Ca_3.1 blocker TRAM-34 (200 nM) [19] (n = 19 asthmatic cells, n = 14 healthy cells) (**Fig 2D**, data in **S10 Table**, and **Fig E**, data in **S11 Table**).

#### Freshly dispersed HBECs from bronchial brushings

**Fig 3:**
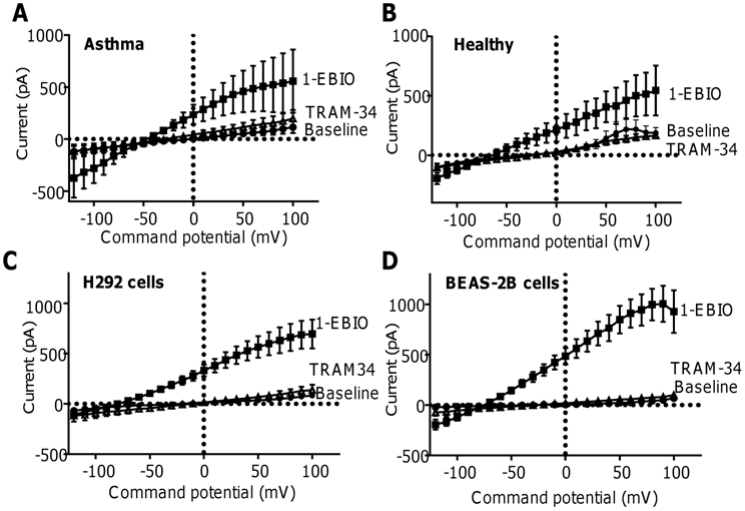
Freshly isolated HBECs, and the H292 and BEAS-2B airway epithelial cell lines exhibit K_Ca_3.1 channel activity. Current-voltage curves demonstrating that 100 μM 1-EBIO induced characteristic K_Ca_3.1 channel currents in freshly isolated HBECs isolated from (**A**) one patient with asthma (n = 3 cells) and (**B**) one healthy control (n = 3 cells). TRAM-34 (200 nM) blocked 1-EBIO-induced currents to near-baseline levels. Characteristic 1-EBIO-dependent K_Ca_3.1 channel activity, sensitive to TRAM-34, was also seen in (**C**) the H292 cell line (n = 6 cells) and (**D**) the BEAS-2B cell line (n = 8 cells).

Addition of 1-EBIO (100 μM) to freshly isolated HBECs obtained from one healthy donor and one asthmatic donor elicited a typical K_Ca_3.1 current in 6 out of 11 cells tested. The number of HBECs responding to 1-EBIO and TRAM-34 were similar in healthy and asthmatic donors; data are summarised in Fig 3A (data in S12 Table) and B (data in S13 Table) and represent recordings from HBECs that responded to both 1-EBIO and subsequently received TRAM-34 (n = 3 asthmatic cells, n = 3 healthy cells).

### BEAS-2B and H292 cell lines

Typical K_Ca_3.1 currents were also induced by 1-EBIO (100 μM) in both H292 cells (n = 6; **Fig 3C**, data in **S14 Table**) and BEAS-2B (n = 8; **Fig 3D**, data in **S15 Table**), and these were inhibited by TRAM-34 (200 nM). In both cell lines, 100% of cells responded to 1-EBIO.

### K_Ca_3.1 channels do not regulate HBEC proliferation or wound healing

Blockade of K_Ca_3.1 channels with TRAM-34 (200 nM) or ICA-17043 (100 nM) did not inhibit the spontaneous proliferation BEAS-2B cells (n = 3 independent experiments in triplicate) or the serum-dependent proliferation of the H292 cell line (n = 4 independent experiments in triplicate; data not shown). Additionally, K_Ca_3.1 blockade by TRAM-34 and ICA-17043 also failed to inhibit primary HBEC wound healing (data not shown).

### K_Ca_3.1 channels do not regulate epithelial mucus synthesis or secretion

**Fig 4:**
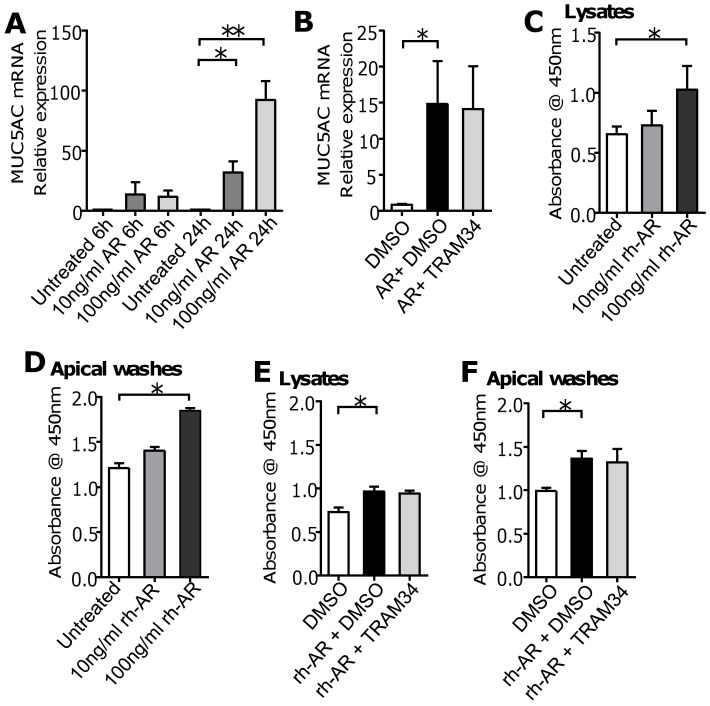
The K_Ca_3.1 channel does not regulate epithelial mucus production or secretion. (**A**) Recombinant human amphiregulin (rh-AR) upregulated MUC5AC mRNA expression in H292 cells cultured in 6 well plates in a concentration-dependent manner after 24 h (*P = 0.010; **P = 0.028; n = 3 individual experiments). (**B**) Pre-treatment of H292 cells with the K_Ca_3.1 blocker TRAM-34 (200 nM) for 30 min prior to stimulation with 10 ng/ml rh-AR for 24 h did not prevent rh-AR-induced MUC5AC mRNA expression (*P = 0.008, **P = 0.042; n = 6 individual experiments). rh-AR dose-dependently increased the mucin content of (**C**) H292 ALI culture lysates (*P = 0.029; n = 3 individual experiments) and (**D**) H292 ALI culture apical washes (*P = 0.009; n = 4 individual experiments) after 24 h, analysed by ELLA. Pre-treatment of H292 ALI cultures with 200 nM TRAM-34 did not prevent rh-AR-induced upregulation of mucin content within (**E**) H292 lysates (*P = 0.019; n = 6 individual experiments) or (**F**) apical washes (*P = 0.027; n = 3 individual experiments). Data are expressed as mean ± SEM.

Amphiregulin is a growth factor implicated in the development of mucus hypersecretion in asthmatic airways [42,43]. qPCR demonstrated that MUC5AC mRNA was expressed in submerged unstimulated H292 monolayers, and was significantly upregulated by recombinant human amphiregulin (rh-AR; R&D Systems) after 24 h in a concentration-dependent manner (10 ng/ml rh-AR: P = 0.010, n = 9; 100 ng/ml rh-AR: P = 0.028, n = 3; **Fig 4A**, data in **S16 Table**). However, pre-treatment of cells with the K_Ca_3.1 blocker, TRAM-34 (200 nM), did not inhibit rh-AR-induced MUC5AC mRNA expression in H292 cell cultures after 24 h (*n* = 6 independent experiments; **Fig 4B**, data in **S17 Table**).

In addition, H292 cells were studied at ALI culture. ELLA analysis of H292 ALI culture lysates revealed that rh-AR dose-dependently increased intracellular mucin content (n = 3 independent experiments; P = 0.029; **Fig 4C**, data in **S18 Table**), and increased the mucin content of apical washes (n = 4 independent experiments; P = 0.009; **Fig 4D**, data in **S19 Table**). However, incubation with TRAM-34 (200 nM) did not reduce either the intracellular mucin content of lysates (**Fig 4E**, data in **S20 Table**) or the mucin content of the apical washes (**Fig 4F**, data in **S21 Table**).

### K_Ca_3.1 channel block does not adversely affect HBEC ciliary beat

**Fig 5:**
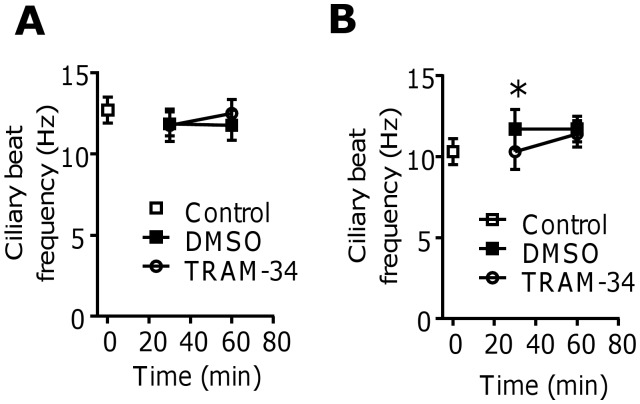
The K_Ca_3.1 channel does not regulate airway epithelial ciliary beat frequency. (**A**) Ciliary beat frequency of epithelial cells from healthy controls treated with either TRAM-34 or DMSO (n = 3). (**B**) Ciliary beat frequency of epithelial cells from asthmatic subjects treated with either TRAM-34 or DMSO (n = 3). *P = 0.001; data are expressed as mean ± SEM.

There was no significant difference in the ciliary beat frequency between TRAM-34 and DMSO treated cilia from healthy volunteers (n = 3) at 30 or 60 min, and no significant change from baseline (**Fig 5A**, data in **S22 Table**). In the cilia from the asthmatic patients (n = 3) there was a very small but statistically significant decrease in the ciliary beat frequency with TRAM-34 (200 nM) treatment compared to DMSO at 30 min (P = 0.011; **Fig 5B**, data in **S23 Table**), but no change from baseline. At 60 min there was no significant difference compared to DMSO control. Each time point in **Fig 5A and 5B** represents the mean ± standard error of 27 ciliary beat frequency measurements from separate cilia. The percentage of immotile and dyskinetic cilia did not change with the addition of TRAM-34 in the healthy or asthmatic cilia (data not shown).

### K_Ca_3.1 block attenuates several features of TGFβ1-dependent epithelial-mesenchymal transition in BEAS-2B cells

**Fig 6:**
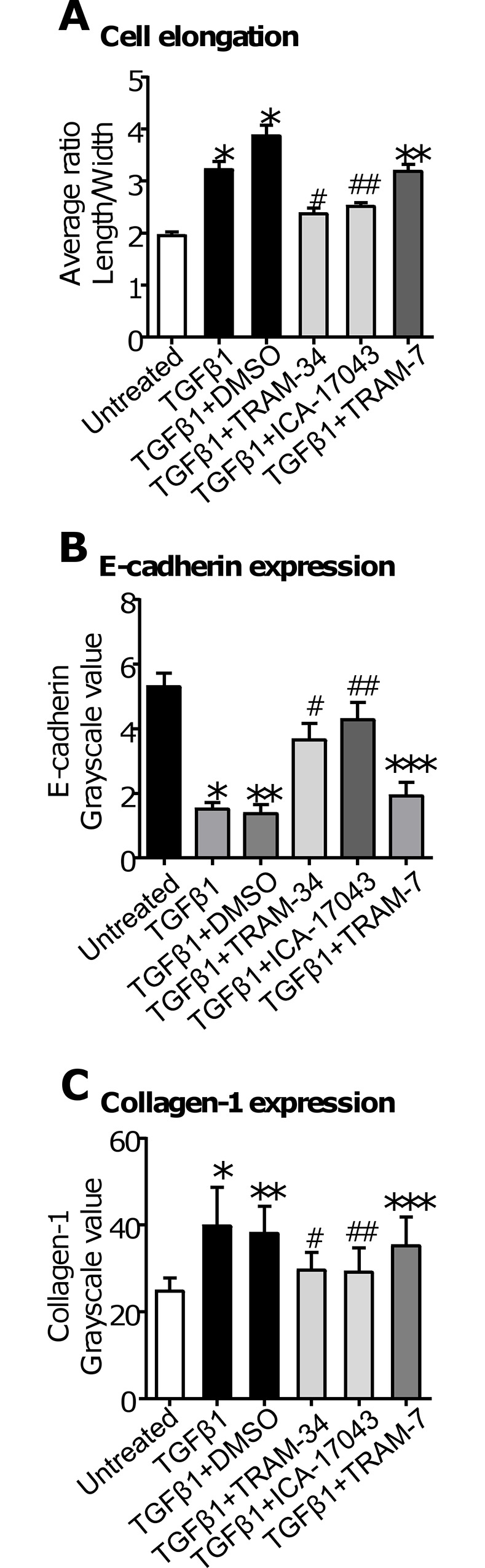
Inhibition of the K_Ca_3.1 channel attenuates features of TGFβ1-dependent EMT. (**A**) Cell elongation, quantified by a ratio of cell length:cell width of vimentin-stained BEAS-2B cells after 72 h, was increased by TGFβ1 or TGFβ1+DMSO compared to untreated cells (0.1% PBS/BSA; *P < 0.001). Pre-treatment of BEAS-2B cells with 200 nM TRAM-34 (#P < 0.001) or 100 nM ICA-17043 (##P = 0.027) significantly inhibited TGFβ1-induced cell elongation compared to TGFβ1+DMSO control. In contrast, TRAM-7, an inactive analog of TRAM-34, did not inhibit TGFβ1-induced cell elongation (**P = 0.022 compared to TRAM-34). Data are presented as mean ± SEM from 6 individual experiments. (**B**) BEAS-2B cells treated with 10 ng/ml TGFβ1 or TGFβ1+DMSO for 72 h exhibited a loss of E-cadherin expression in comparison to untreated cells (0.1% PBS/BSA; *P < 0.001, **P = 0.001). Pre-treatment with TRAM-34 (200 nM; #P = 0.013) or ICA-17043 (100 nM; ##P = 0.002) attenuated the TGFβ1-dependent loss of E-cadherin compared to TGFβ1+DMSO. TRAM-7 (200 nM) did not prevent TGFβ1-induced loss of E-cadherin immunostaining (***P = 0.014 compared to TRAM-34). Data are presented as mean ± SEM from 6 individual experiments. (**C**) BEAS-2B cells treated with 10 ng/ml TGFβ1 or 10 ng/ml TGFβ1+DMSO control for 72 h displayed an increase in collagen-1 expression in comparison to untreated cells (0.1% PBS/BSA) (*P = 0.005; **P = 0.028 respectively). However, pre-treatment with TRAM-34 (200 nM; #P = 0.021) or ICA-17043 (100 nM; ##P = 0.024) significantly inhibited this effect compared to TGFβ1+DMSO. TRAM-7 (200 nM) did not inhibit TGFβ1-induced upregulation of collagen-1 immunostaining (***P = 0.021 compared to ICA-17043). Data are presented as mean ± SEM from 5 individual experiments.

Incubation of BEAS-2B cells with TGFβ1 (10 ng/ml) for 72 h induced a marked change in cell morphology; cells exhibited both loss of the typical cobblestone appearance and developed an elongated fibroblastoid appearance (**Fig A in S3 Fig**). To quantify this, the ratio of length/width of vimentin-stained cells was calculated. TGFβ1 caused a significant increase in the length/width ratio (indicating elongation of cells) in comparison with unstimulated cells (P < 0.001; n = 6 **Fig 6A**, data in **S24 Table**). However, K_Ca_3.1 blockade attenuated this change in morphology (**Fig 6A, Fig B in S3 Fig**). In comparison with cells treated with TGFβ1 and DMSO (0.1%, vehicle control), treatment with either TRAM-34 (200 nM; P < 0.001) or ICA-17043 (100 nM; P = 0.027) significantly inhibited this TGFβ1-induced increase in length/width ratio (**Fig 6A**). An inactive analog of TRAM-34, TRAM-7 (200 nM), did not inhibit TGFβ1-dependent shape change (P = 0.022 compared to TRAM-34; **Fig 6A**).

Another key step in the development of EMT is the loss of E-cadherin expression. TGFβ1-induced cell elongation was accompanied by a marked loss in immunofluorescent E-cadherin staining after 72 h (P < 0.001; n = 6; **Fig 6B**, data in **S25 Table**, and **Fig C in S3 Fig**). This loss of E-cadherin expression was significantly attenuated by both TRAM-34 (200 nM; P = 0.013; **Fig 6B**) and ICA-17043 (100 nM; P = 0.02; **Fig 6B**), but not TRAM-7 (P = 0.014 in comparison to TRAM-34; **Fig 6B**).

Previous studies showed that TGFβ1 increases vimentin protein expression in BEAS-2B cells, detected by western blot [44]. However we found that vimentin was readily detectable in both resting BEAS-2B cells, and in cells stimulated with TGFβ1. Consequently, no obvious differences were discernible for this “fibroblast marker”. However, in accordance with previous studies, we have found TGFβ1-induced upregulation of collagen-1 immunofluorescence staining in BEAS-2B cells (TGFβ1: P = 0.005; TGFβ1 with 0.1% DMSO, vehicle control: P = 0.028; n = 5; **Fig 6C**, data in **S26 Table**, and **Fig D in S3 Fig**). Data are expressed as a fold change above baseline (cells treated with 0.1% PBS/BSA alone; where baseline = 1). Treatment with 200nM TRAM-34 (P = 0.021) or 100 nM ICA-17043 (P = 0.024) significantly inhibited this TGFβ1-induced collagen-1 expression (**Fig 6C**). TRAM-7 (200 nM) did not attenuate TGFβ1-induced collagen-1 immunofluorescence staining (P = 0.021 in comparison with ICA-17043; **Fig 6C**).

## Discussion

The intermediate conductance Ca^2+^-activated K^+^ channel K_Ca_3.1 inhibits the proliferation and migration of several cell types, effects which may be detrimental if applicable to the damaged airway epithelium in diseases such as asthma and idiopathic pulmonary fibrosis. We have therefore examined in detail the expression and function of K_Ca_3.1 in primary human bronchial epithelial cells (HBECs) and airway epithelial cell lines (H292 and BEAS-2B). The key finding from our study is that primary HBECs express K_Ca_3.1 both *in vitro* and *in vivo*. Blocking this channel did not adversely epithelial cell function, but inhibited several aspects of TGFβ1-dependent EMT. This supports the view that targeting K_Ca_3.1 may be beneficial for the treatment of pulmonary disease [7], and safe from the perspective of the airway epithelium.

K_Ca_3.1 expression has been described previously in HBEC cell lines and transformed cells [13–15], and based on pharmacological studies, was probably present in cultured primary HBECs [18]. We show that primary HBECs express K_Ca_3.1 mRNA and protein, the latter both *in vitro* and *ex vivo*. Importantly, functional K_Ca_3.1 channels revealed by patch clamp analysis were present in the plasma membrane of both cultured and freshly dispersed primary HBECs. K_Ca_3.1 channels were expressed in HBECs from both healthy control subjects and a disease group with asthma. K_Ca_3.1 whole cell currents were larger in the cells from asthmatic subjects, despite similar levels of mRNA and protein expression. The biological significance of this requires further investigation.

K_Ca_3.1 activity has been shown to be important for the migration of several cell types including mast cells [6], glial cells [45], monocyte/macrophages [46], NIH3T3 fibroblasts [47], fibrocytes [39], melanoma cells [47] and epithelial cells [48]. The mechanism is not clear, but it is proposed that K_Ca_3.1 activity is required for detachment of the uropod, through the regulation of cell volume and actin dynamics [49]. It might be expected therefore that K_Ca_3.1 inhibits the ability of HBECs to migrate into and subsequently heal a wound. Since there is an abnormality of epithelial wound healing in asthma [12], any such effect might exacerbate rather than attenuate asthma immunopathology. Trinh et al described the inhibition of wound healing by transformed HBECs (NuLi and CuFi) using very high concentrations of TRAM-34 (5 μM) [15], but which are non-selective at this concentration [7]. The K_d_ of TRAM-34 is 20 nM, and at 5–10 times the K_d_, nearly all channels will be blocked [50]. Using relevant and selective concentrations of TRAM-34 (20–200 nM) we saw no effect of K_Ca_3.1 block on HBEC wound healing or proliferation, suggesting that K_Ca_3.1 does not regulate these processes, and so adverse effects on this aspect of airway biology are unlikely to occur with treatment *in vivo*.

K_Ca_3.1 activity has also been proposed to play an important role in airway ion transport. In Calu-3 cells K_Ca_3.1 serves is to reduce HCO^3-^ secretion and increase Cl^-^ secretion, and hence promote airway surface liquid hydration. It has therefore been suggested that K_Ca_3.1 openers might be useful in the treatment of cystic fibrosis [14]. K_Ca_3.1 block might therefore exert unwanted effects in airway diseases such as asthma if it reduces airway hydration. However, ALI morphology, ciliary function and mucus production and composition were not altered by K_Ca_3.1 blockade, suggesting that airway secretion and clearance will not be altered by K_Ca_3.1 treatment *in vivo*. Such an absence of adverse effects on HBECs *in vitro* is supported by *in vivo* data. Clinical trials of K_Ca_3.1 blockers in humans have not reported any increase in respiratory system side effects, and K_Ca_3.1 knockout mice and animals treated with TRAM-34 for many weeks demonstrate a normal respiratory phenotype [7]. Furthermore, K_Ca_3.1 blockade was efficacious in both mouse and sheep models of asthma [8,9].

K_Ca_3.1 is thought to play a key role in the development of fibrosis due to its roles in renal fibroblast proliferation and fibrogenesis [51], and also human lung myofibroblast proliferation, wound healing, collagen secretion and contraction [40,52,53]. TGFβ1, a prominent mediator of fibrotic diseases, has been shown to increase myofibroblast K_Ca_3.1 mRNA expression, and its ability to induce pro-fibrotic function is associated with K_Ca_3.1-dependent regulation of intracellular Ca^2+^ signalling [40]. Importantly, TGFβ1 is also thought to mediate EMT, a process implicated in the formation of scars following epithelial damage, and with potential roles in fibrosis in following tissue injury [54–56]. Diseases such as COPD [57–59], IPF [56] and lung cancer [60,61] display features of EMT, and histological analysis of asthmatic airways has found subepithelial fibrosis and hyperplasia of myofibroblasts in close proximity to the basement membrane [62]. Consequently, understanding the mechanisms driving fibrotic changes in asthma could provide insight into the dysfunction of the asthmatic airway epithelium. Our data indicate that the K_Ca_3.1 channel may play a key role in the initial stages of TGFβ1-dependent EMT, as shown by inhibition of shape change and loss of E-cadherin protein expression with K_Ca_3.1 blockade. We also found that K_Ca_3.1 blockade prevented the upregulation of collagen-1 expression by TGFβ1, which corresponds with previous work in human lung myofibroblasts [40,53]. If K_Ca_3.1 blockade inhibits three key and early steps in the process of EMT *in vivo*, K_Ca_3.1 blockade may not only be useful for the inhibition of lung parenchymal and airway wall fibrosis, but also the prevention of cancer progression.

In summary we have shown that HBECs express the K^+^ channel K_Ca_3.1, but blocking this channel *in vitro* does not alter HBEC wound healing, proliferation, mucus secretion or ciliary function. As such, treatment of diseases such as asthma and pulmonary fibrosis with K_Ca_3.1 blockers is unlikely to have adverse effects on airway biology. Of note, K_Ca_3.1 blockade reduced several aspects of TGFβ1-dependent EMT, suggesting that K_Ca_3.1 blockers may reduce airway wall fibrosis in asthma and COPD.

## Supporting Information

S1 Fig Data for Fig 1C. Original uncropped western blots for K_Ca_3.1 and β-actin
(PDF)

S2 Fig K_Ca_3.1 and MUC5AC expression are upregulated in the severe asthmatic bronchial epithelium. (**A**) K_Ca_3.1 protein expression and (**B**) MUC5AC expression were detected by immunostaining in the airway epithelium of GMA-embedded biopsies isolated from patients with severe, moderate or mild asthma and healthy controls. An increase in staining for both was seen in the severe asthmatic bronchial epithelium in comparison to the mild asthmatic and healthy control bronchial epithelium.
(TIF)

S3 Fig K_Ca_3.1 channel blockade prevents TGFβ1-induced epithelial-mesenchymal transition. (**A**) BEAS-2B cells treated with 10 ng/ml TGFβ1 for 72 h and stained with FITC-conjugated anti-vimentin antibody exhibited elongation in comparison to untreated cells (0.1% PBS/BSA). Staining with DAPI was used to visualise cell nuclei. (**B**) Pre-treatment of BEAS-2B cells with TRAM-34 (200 nM) or ICA-17043 (100 nM) to block K_Ca_3.1 channel activity inhibited TGFβ1-induced elongation of the vimentin-stained cells after 72 h. (**C**) BEAS-2B cells treated with 10 ng/ml TGFβ1 for 72 h and stained with FITC-conjugated anti-E-cadherin antibody exhibited a loss of E-cadherin expression in comparison to untreated cells. TRAM-34 (200 nM) and ICA-17043 (100 nM), but not TRAM-7 (200 nM), inhibited TGFβ1-induced down-regulation of E-cadherin expression after 72 h. (**D**) 10 ng/ml TGFβ1 for 72 h upregulated collagen-1 expression in BEAS-2B cells, visualised by staining with FITC-conjugated anti-collagen-1 antibody, in comparison to untreated cells. ICA-17043 (100 nM) and TRAM-34 (200 nM) inhibited TGFβ1-induced upregulation of collagen-1 expression after 72 h. TGFb1-dependent collagen upregulation was not altered by TRAM-7 (200 nM).
(TIF)

S1 Table Data for Fig 1B. ΔCT scores expressed as transcripts/10^3^ β-actin of PCR reactions with K_Ca_3.1 primers.
(PDF)

S2 Table Data for Fig 1D. Immunostaining values (expressed as percentages) of Cell^F^ analysis of bronchial biopsy specimens stained with anti-K_Ca_3.1 antibody.
(PDF)

S3 Table Data for Fig 1E. Immunostaining values (expressed as percentages) of Cell^F^ analysis of bronchial biopsy specimens stained with anti-K_Ca_3.1 antibody.
(PDF)

S4 Table Data for Fig 1F. Number of MUC5AC-positive cells expressing K_Ca_3.1 immunostaining (expressed as percentages).
(PDF)

S5 Table Data for Fig 1G. Area fraction values (expressed as percentages) of Cell^F^ analysis of bronchial biopsy specimens stained with anti-MUC5AC antibody.
(PDF)

S6 Table Data for Fig 1H. Area fraction values (expressed as percentages) of Cell^F^ analysis of bronchial biopsy specimens stained with anti-MUC5AC antibody.
(PDF)

S7 Table Data for Fig 1I. Area fraction values of Cell^F^ analysis of bronchial biopsy specimens stained with anti-K_Ca_3.1 and anti-MUC5AC antibodies.
(PDF)

S8 Table Data for Fig 2A. Current values and command potential (mV) values for currents recorded from asthmatic and healthy primary HBECs at baseline.
(PDF)

S9 Table Data for Fig 2B. 1-EBIO-dependent current values plotted against command potential values recorded from asthmatic and healthy primary HBECs.
(PDF)

S10 Table Data for Fig 2D. Current values plotted against command potential (mV) values for currents recorded at baseline, and following the sequential addition of 1-EBIO and TRAM-34 from asthmatic HBECs.
(PDF)

S11 Table Data for Fig 2E. Current values plotted against command potential (mV) values for currents recorded at baseline, and following the sequential addition of 1-EBIO and TRAM-34 from healthy HBECs.
(PDF)

S12 Table Data for Fig 3A. Current values plotted against command potential (mV) values for currents recorded at baseline, and following the sequential addition of 1-EBIO and TRAM-34 from freshly brushed asthmatic HBECs.
(PDF)

S13 Table Data for Fig 3B. Current values plotted against command potential (mV) values for currents recorded at baseline, and following the sequential addition of 1-EBIO and TRAM-34 from freshly brushed healthy HBECs.
(PDF)

S14 Table Data for Fig 3C. Current values plotted against command potential (mV) values for currents recorded at baseline, and following the sequential addition of 1-EBIO and TRAM-34 from H292 cells.
(PDF)

S15 Table Data for Fig 3D. Current values plotted against command potential (mV) values for currents recorded at baseline, and following the sequential addition of 1-EBIO and TRAM-34 from BEAS-2B cells.
(PDF)

S16 Table Data for Fig 4A. ΔCT scores expressed as transcripts/10^6^ 18S mRNA of PCR reactions with MUC5AC TaqMan probes.
(PDF)

S17 Table Data for Fig 4B. ΔCT scores expressed as transcripts/10^6^ 18S mRNA of PCR reactions with MUC5AC TaqMan probes.
(PDF)

S18 Table Data for Fig 4C. Absorbance values detected at 450 nm.
(PDF)

S19 Table Data for Fig 4D. Absorbance values detected at 450 nm.
(PDF)

S20 Table Data for Fig 4E. Absorbance values detected at 450 nm.
(PDF)

S21 Table Data for Fig 4F. Absorbance values detected at 450 nm.
(PDF)

S22 Table Data for Fig 5A. Ciliary beat frequency (Hz) of epithelial cells from healthy controls.
(PDF)

S23 Table Data for Fig 5B. Ciliary beat frequency (Hz) of epithelial cells from asthmatic donors.
(PDF)

S24 Table Data for Fig 6A. Values of ratio of cell length:cell width of vimentin-stained BEAS-2B cells.
(PDF)

S25 Table Data for Fig 6B. Grayscale values of E-cadherin-stained BEAS-2B cells.
(PDF)

S26 Table Data for Fig 6C. Grayscale values of collagen-1-stained BEAS-2B cells.
(PDF)

## Abbreviations

HLMC: human lung mast cell
HBEC: human bronchial epithelial cell
1-EBIO: 1-ethyl-2-benzimidazolinone
V_m_: whole cell current reversal potential
rh-AR: recombinant human amphiregulin
