# Supplementary material for: KCa3.1 K+ Channel Expression and Function in Human Bronchial Epithelial Cells
Source: PLoS One. 2015 Dec 21;10(12):e0145259. doi: 10.1371/journal.pone.0145259 (PMC4687003; doi:10.1371/journal.pone.0145259)

Original uncropped blots for Figure 1C

Western blot with anti-K_{Ca}3.1 antibody (short exposure to show protein ladder):

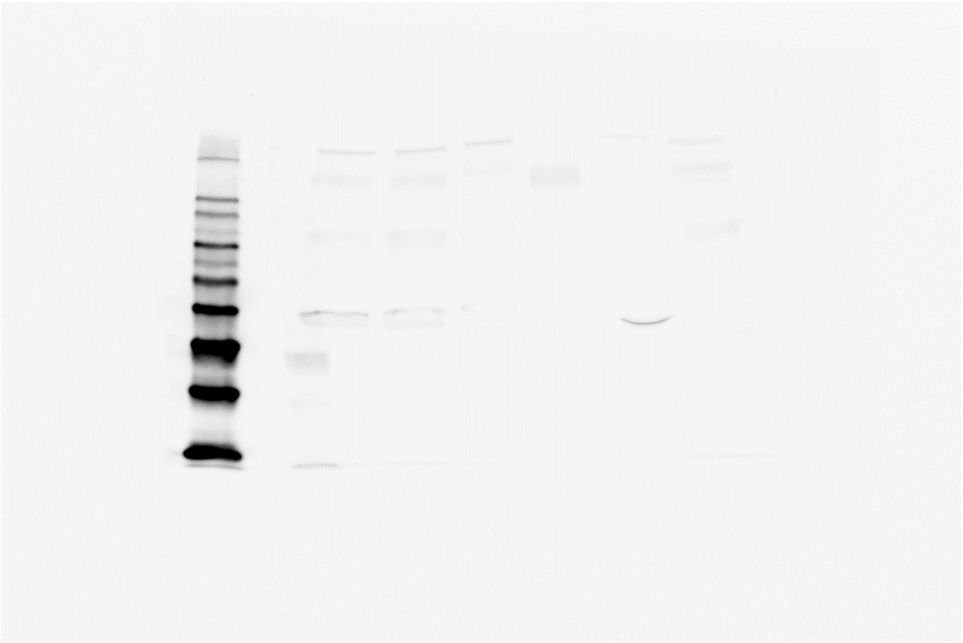

Western blot with anti-K_{Ca}3.1 antibody (longer exposure to show target bands):

Lanes:

Ladder; donor A026; donor A027; donor NA001.

Last 3 lanes = other samples, not applicable to this study.

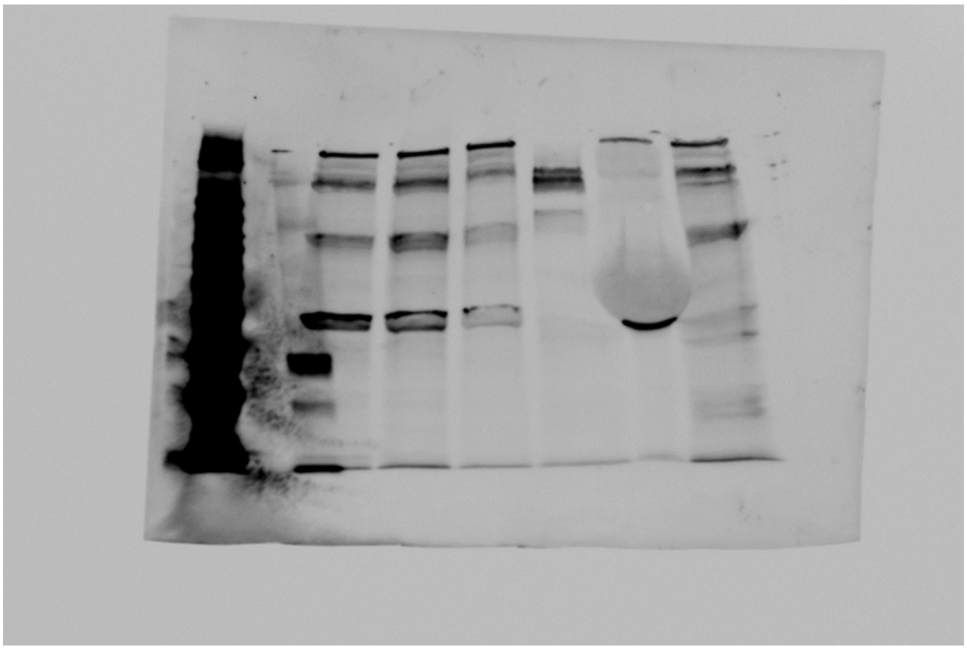

Western blot with anti-β-actin antibody after membrane was stripped to remove anti-$K_{Ca}3.1$ antibody:

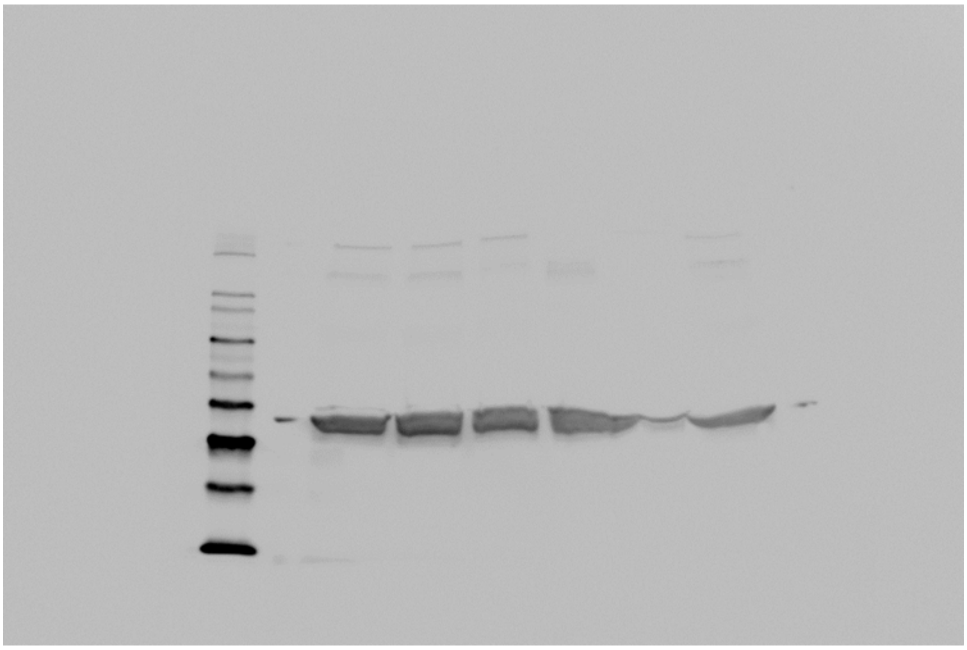

Supplement: S1 Fig — Original uncropped western blots for KCa3.1 and β-actin (PDF) [file pone.0145259.s001.pdf]
