# Supplementary figures and images for: KCa3.1 K+ Channel Expression and Function in Human Bronchial Epithelial Cells
Source: PLoS One. 2015 Dec 21;10(12):e0145259. doi: 10.1371/journal.pone.0145259 (PMC4687003; doi:10.1371/journal.pone.0145259)

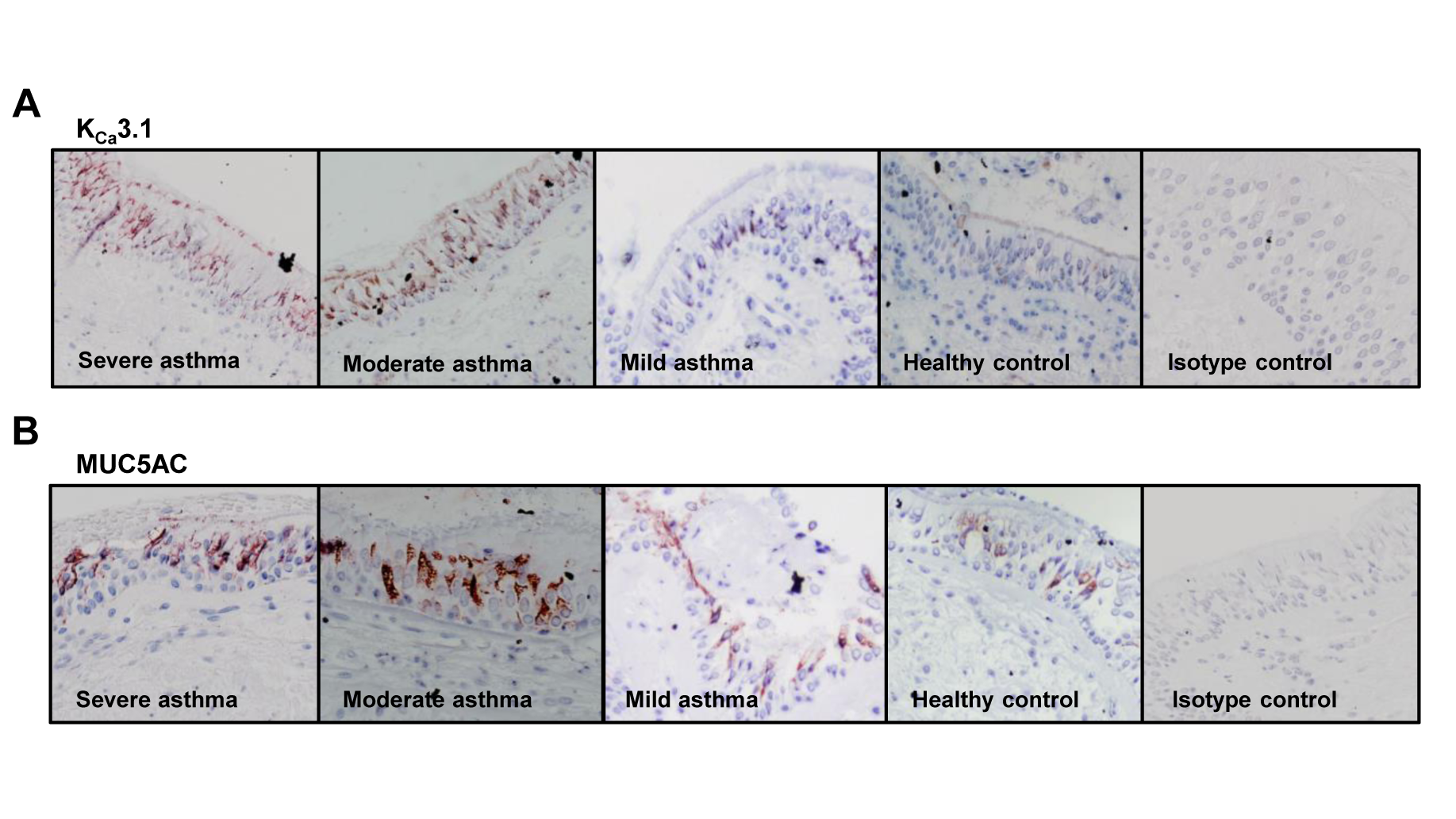

Supplement: S2 Fig — (A) KCa3.1 protein expression and (B) MUC5AC expression were detected by immunostaining in the airway epithelium of GMA-embedded biopsies isolated from patients with severe, moderate or mild asthma and healthy controls. An increase in staining for both was seen in the severe asthmatic bronchial epithelium in comparison to the mild asthmatic and healthy control bronchial epithelium. (TIF) [file pone.0145259.s002.tif]

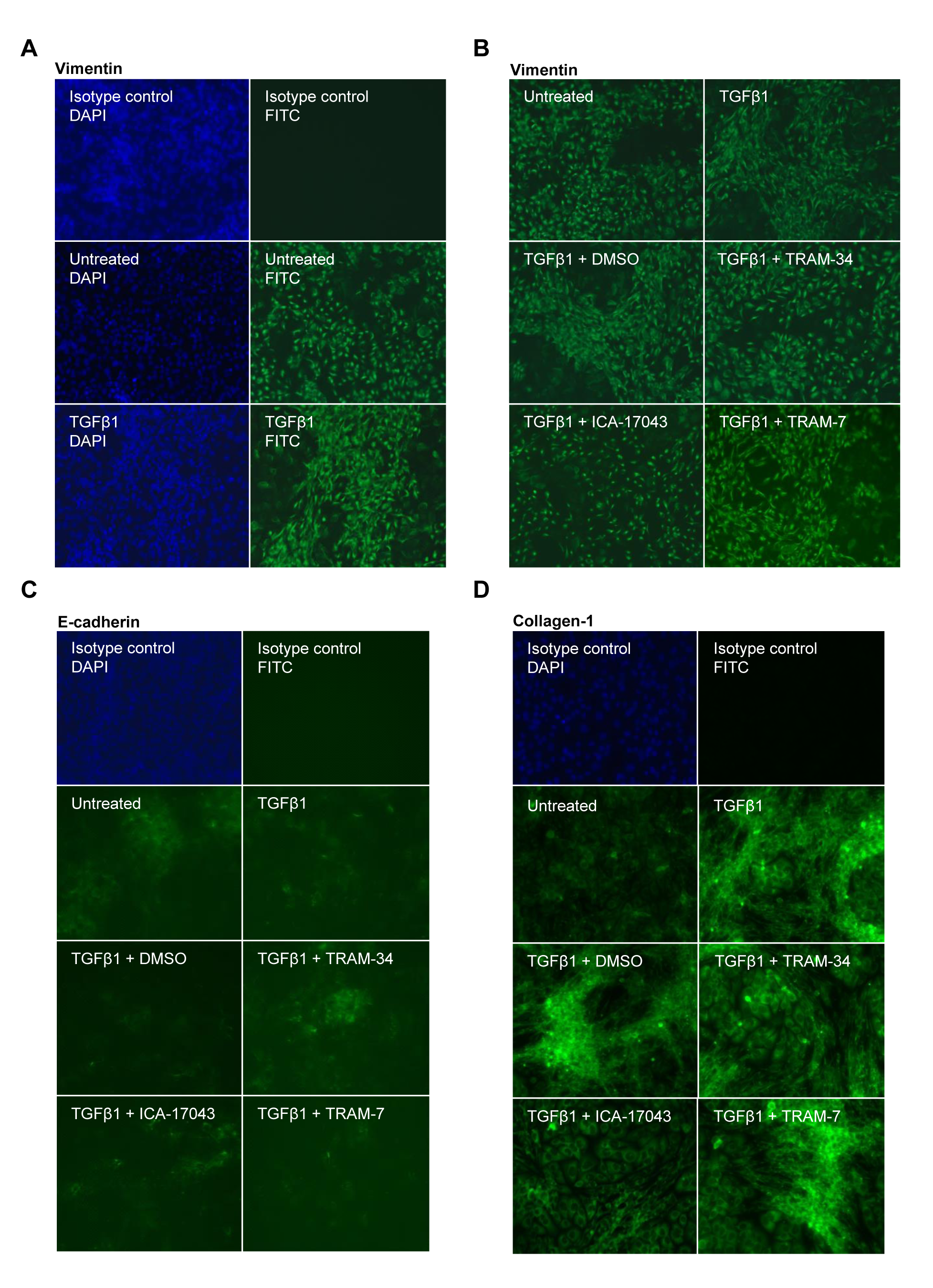

Supplement: S3 Fig — (A) BEAS-2B cells treated with 10 ng/ml TGFβ1 for 72 h and stained with FITC-conjugated anti-vimentin antibody exhibited elongation in comparison to untreated cells (0.1% PBS/BSA). Staining with DAPI was used to visualise cell nuclei. (B) Pre-treatment of BEAS-2B cells with TRAM-34 (200 nM) or ICA-17043 (100 nM) to block KCa3.1 channel activity inhibited TGFβ1-induced elongation of the vimentin-stained cells after 72 h. (C) BEAS-2B cells treated with 10 ng/ml TGFβ1 for 72 h and stained with FITC-conjugated anti-E-cadherin antibody exhibited a loss of E-cadherin expression in comparison to untreated cells. TRAM-34 (200 nM) and ICA-17043 (100 nM), but not TRAM-7 (200 nM), inhibited TGFβ1-induced down-regulation of E-cadherin expression after 72 h. (D) 10 ng/ml TGFβ1 for 72 h upregulated collagen-1 expression in BEAS-2B cells, visualised by staining with FITC-conjugated anti-collagen-1 antibody, in comparison to untreated cells. ICA-17043 (100 nM) and TRAM-34 (200 nM) inhibited TGFβ1-induced upregulation of collagen-1 expression after 72 h. TGFb1-dependent collagen upregulation was not altered by TRAM-7 (200 nM). (TIF) [file pone.0145259.s003.tif]
