# Supplementary material for: KCa3.1 K+ Channel Expression and Function in Human Bronchial Epithelial Cells
Source: PLoS One. 2015 Dec 21;10(12):e0145259. doi: 10.1371/journal.pone.0145259 (PMC4687003; doi:10.1371/journal.pone.0145259)
Supplement: S1 Table — ΔCT scores expressed as transcripts/103 β-actin of PCR reactions with KCa3.1 primers. (PDF) [file pone.0145259.s004.pdf]

Asthmatics	Healthy controls
3.06	0.55
1.08	0.64
1.64	1.22
0.68	1.98
0.96	1.64
1.55	
1.47	
1.9	
0.74	
0.48	
