# Supplementary material for: KCa3.1 K+ Channel Expression and Function in Human Bronchial Epithelial Cells
Source: PLoS One. 2015 Dec 21;10(12):e0145259. doi: 10.1371/journal.pone.0145259 (PMC4687003; doi:10.1371/journal.pone.0145259)
Supplement: S2 Table — Immunostaining values (expressed as percentages) of CellF analysis of bronchial biopsy specimens stained with anti-KCa3.1 antibody. (PDF) [file pone.0145259.s005.pdf]

Asthmatics	Healthy controls
3.7	0.34
4.97	1.35
6.12	2.54
12.12	0.43
2.66	0.49
2.44	0.06
3.39	0.34
2.79	0.48
4.01	
1.6	
2.55	
0.46	
0.46	
2.26	
1.19	
3.64	
0.68	
1.83	
0.55	
0.14	
0.18	
2.78	
