# Supplementary material for: KCa3.1 K+ Channel Expression and Function in Human Bronchial Epithelial Cells
Source: PLoS One. 2015 Dec 21;10(12):e0145259. doi: 10.1371/journal.pone.0145259 (PMC4687003; doi:10.1371/journal.pone.0145259)
Supplement: S4 Table — Number of MUC5AC-positive cells expressing KCa3.1 immunostaining (expressed as percentages). (PDF) [file pone.0145259.s007.pdf]

Severe asthma	Moderate asthma	Mild asthma	Healthy
40.4	40.5	39.5	49.2
54.8	46.2	56.2	50.2
42.8	43	31.2	49.2
54.6	53.5		35.8
61.3	38.6		23.3
50.5	33.3		46
44.5	56.3		42.1
28.3			20
27.9			
50.8			
44.1			
62.5			
