# Supplementary material for: KCa3.1 K+ Channel Expression and Function in Human Bronchial Epithelial Cells
Source: PLoS One. 2015 Dec 21;10(12):e0145259. doi: 10.1371/journal.pone.0145259 (PMC4687003; doi:10.1371/journal.pone.0145259)
Supplement: S5 Table — Area fraction values (expressed as percentages) of CellF analysis of bronchial biopsy specimens stained with anti-MUC5AC antibody. (PDF) [file pone.0145259.s008.pdf]

Asthmatics	Healthy controls
9.2	0.94
8.86	1
2.36	5.42
8.4	2.19
6.49	0.14
1.13	0.04
0.95	0.67
1.98	0.17
3.31	
4.82	
5.35	
0.38	
0.71	
2.43	
2.04	
3.69	
0.5	
0.5	
7.18	
0.62	
4.71	
1.22	
