# Supplementary material for: KCa3.1 K+ Channel Expression and Function in Human Bronchial Epithelial Cells
Source: PLoS One. 2015 Dec 21;10(12):e0145259. doi: 10.1371/journal.pone.0145259 (PMC4687003; doi:10.1371/journal.pone.0145259)
Supplement: S6 Table — Area fraction values (expressed as percentages) of CellF analysis of bronchial biopsy specimens stained with anti-MUC5AC antibody. (PDF) [file pone.0145259.s009.pdf]

Severe asthmatics	Moderate asthmatics	Mild asthmatics	Healthy controls
9.2	0.71	7.18	0.94
8.86	2.43	0.62	1
2.36	2.04	4.71	5.42
8.4	3.69		2.19
6.49	0.5		0.14
1.13	0.5		0.04
0.95	1.22		0.67
1.98			0.17
3.31			
4.82			
5.35			
0.38			
