# Supplementary material for: KCa3.1 K+ Channel Expression and Function in Human Bronchial Epithelial Cells
Source: PLoS One. 2015 Dec 21;10(12):e0145259. doi: 10.1371/journal.pone.0145259 (PMC4687003; doi:10.1371/journal.pone.0145259)
Supplement: S7 Table — Area fraction values of CellF analysis of bronchial biopsy specimens stained with anti-KCa3.1 and anti-MUC5AC antibodies. (PDF) [file pone.0145259.s010.pdf]

MUC5AC	KCa3.1
9.2	3.7
8.86	4.97
2.36	6.12
8.4	12.12
6.49	2.66
1.13	2.44
0.95	3.39
1.98	2.79
3.31	4.01
4.82	1.6
5.35	2.55
0.38	0.46
7.18	0.55
0.62	0.14
4.71	0.18
0.71	0.46
2.43	2.26
2.04	1.19
3.69	3.64
0.5	0.68
0.5	1.83
0.94	0.34
1	1.35
5.42	2.54
2.19	0.43
0.14	0.49
0.04	0.06
0.67	0.34
0.17	0.48
1.22	2.78
