# Supplementary material for: KCa3.1 K+ Channel Expression and Function in Human Bronchial Epithelial Cells
Source: PLoS One. 2015 Dec 21;10(12):e0145259. doi: 10.1371/journal.pone.0145259 (PMC4687003; doi:10.1371/journal.pone.0145259)
Supplement: S8 Table — Current values and command potential (mV) values for currents recorded from asthmatic and healthy primary HBECs at baseline. (PDF) [file pone.0145259.s011.pdf]

Command potential (mV)	Asthmatics (n = 20)		Non-asthmatics (n = 14)	
-120	-43.27	7.62	-45.18	7.56
-110	-38.29	7.09	-39.11	6.58
-100	-33.65	6.09	-34.02	5.54
-90	-27.9	5.26	-29.47	5.14
-80	-23.05	4.38	-24.87	4.99
-70	-19.34	4.09	-20.05	4.65
-60	-14.69	3.25	-17.6	4.29
-50	-10.58	2.99	-13.75	3.77
-40	-6.68	3.12	-9.58	3.41
-30	-1.97	3.41	-5.21	3.17
-20	2.93	4.57	-1.95	2.94
-10	7.73	5.29	1.73	2.51
0	13.69	7.65	6.79	2.82
10	20.03	8.93	11.6	2.85
20	24.13	9.23	17.63	3.53
30	33.27	11.99	22.56	4.14
40	39.66	11.59	27.36	5.41
50	48.73	12.51	35.87	7.1
60	63.96	15.61	45.97	9.14
70	75.77	14.85	57.81	11.35
80	101.72	19.08	72.21	14.82
90	137.99	23.39	91.46	18.07
100	197.49	31.43	118.6	22.96
