# Supplementary material for: KCa3.1 K+ Channel Expression and Function in Human Bronchial Epithelial Cells
Source: PLoS One. 2015 Dec 21;10(12):e0145259. doi: 10.1371/journal.pone.0145259 (PMC4687003; doi:10.1371/journal.pone.0145259)
Supplement: S9 Table — 1-EBIO-dependent current values plotted against command potential values recorded from asthmatic and healthy primary HBECs. (PDF) [file pone.0145259.s012.pdf]

Command potential (mV)	Asthma		Healthy	
-120	-102.72	25.88	-19.9	5.76
-110	-93.97	22.68	-20.68	5.92
-100	-80.65	20	-15.81	3.99
-90	-65.34	16.33	-10.61	3.88
-80	-49.61	13.77	-5.57	4.09
-70	-33.46	12.5	0.55	4.38
-60	-15.28	12.99	9.91	5.18
-50	9.4	14.01	18.35	6.03
-40	35.1	16.4	26.11	7.77
-30	61.07	19.87	36.78	9.29
-20	91.35	23.83	45.42	11.52
-10	126.1	28.53	56.06	12.79
0	159.9	32.73	63.5	14.46
10	195.44	38.03	69.51	16.6
20	233.84	44.44	69	17.09
30	276.76	55.22	72.03	17.75
40	317.29	62.81	70.08	17.1
50	343.8	68.06	71.06	17.53
60	363.6	67.39	72.21	16.74
70	395.53	73.05	74.23	17.36
80	420.91	76.44	87.97	20.79
90	438.25	82.03	107.18	28.18
100	451.67	89.88	140.53	35.36
