# Supplementary material for: KCa3.1 K+ Channel Expression and Function in Human Bronchial Epithelial Cells
Source: PLoS One. 2015 Dec 21;10(12):e0145259. doi: 10.1371/journal.pone.0145259 (PMC4687003; doi:10.1371/journal.pone.0145259)
Supplement: S10 Table — Current values plotted against command potential (mV) values for currents recorded at baseline, and following the sequential addition of 1-EBIO and TRAM-34 from asthmatic HBECs. (PDF) [file pone.0145259.s013.pdf]

Command potential (mV)	Baseline		1-EBIO		TRAM-34	
-120	-43.27	7.62	-125.23	36.73	-99.27	41.32
-110	-38.29	7.09	-112.28	31.01	-89.55	37.17
-100	-33.65	6.09	-93.04	25.28	-77.71	33.03
-90	-27.9	5.26	-74.37	19.61	-68.48	29.49
-80	-23.05	4.38	-56.02	14.7	-59.19	25.27
-70	-19.34	4.09	-35.46	11.17	-51.1	21.71
-60	-14.69	3.25	-12.99	11.42	-41.56	17.42
-50	-10.58	2.99	13.36	15.04	-32.5	13.01
-40	-6.68	3.12	40.27	20.46	-24.08	9.51
-30	-1.97	3.41	69.34	27.43	-15.22	5.92
-20	2.93	4.57	104.19	34.41	-6.69	3.46
-10	7.73	5.29	139.95	42.02	2.61	4.45
0	13.69	7.65	176.67	49.43	11.82	7.51
10	20.03	8.93	218.66	57.72	22.04	11.04
20	24.13	9.23	259.14	65.95	31.05	14.34
30	33.27	11.99	313.29	83.72	40.87	17.54
40	39.66	11.59	357.53	93.4	52.34	19.89
50	48.73	12.51	394.22	98.51	64.09	21.64
60	63.96	15.61	418.97	95.61	81.16	24.53
70	75.77	14.85	469.97	100.85	104.84	27.74
80	101.72	19.08	523.23	105.11	141	32.49
90	137.99	23.39	585.11	110.7	185.93	39.37
100	197.49	31.43	684.68	124.09	257.68	46.44
