# Supplementary material for: KCa3.1 K+ Channel Expression and Function in Human Bronchial Epithelial Cells
Source: PLoS One. 2015 Dec 21;10(12):e0145259. doi: 10.1371/journal.pone.0145259 (PMC4687003; doi:10.1371/journal.pone.0145259)
Supplement: S11 Table — Current values plotted against command potential (mV) values for currents recorded at baseline, and following the sequential addition of 1-EBIO and TRAM-34 from healthy HBECs. (PDF) [file pone.0145259.s014.pdf]

| Command potential (mV) | Baseline | | 1-EBIO | | TRAM-34 | |
|---|---|---|---|---|---|---|
| -120 | -45.18 | 7.56 | -66.19 | 7.76 | -49.3 | 5.21 |
| -110 | -39.11 | 6.58 | -60.42 | 7.04 | -42.94 | 4.96 |
| -100 | -34.02 | 5.54 | -50.82 | 5.74 | -38.87 | 4.68 |
| -90 | -29.47 | 5.14 | -40.93 | 4.92 | -33.34 | 4.32 |
| -80 | -24.87 | 4.99 | -29.72 | 3.05 | -28.69 | 3.68 |
| -70 | -20.05 | 4.65 | -18.56 | 2.37 | -24.37 | 3.18 |
| -60 | -17.6 | 4.29 | -6.84 | 2.58 | -19.95 | 2.96 |
| -50 | -13.75 | 3.77 | 6.7 | 3.96 | -17.54 | 2.44 |
| -40 | -9.58 | 3.41 | 20.06 | 6.2 | -11.15 | 2.28 |
| -30 | -5.21 | 3.17 | 34.8 | 8.26 | -8.66 | 2.09 |
| -20 | -1.95 | 2.94 | 48.37 | 11.02 | -3.4 | 2.12 |
| -10 | 1.73 | 2.51 | 62.72 | 13.16 | 0.64 | 2.19 |
| 0 | 6.79 | 2.82 | 75.12 | 15.27 | 5.16 | 2.64 |
| 10 | 11.6 | 2.85 | 86.36 | 18.37 | 9.81 | 2.71 |
| 20 | 17.63 | 3.53 | 93.23 | 19.07 | 16.3 | 3.45 |
| 30 | 22.56 | 4.14 | 101.51 | 20.28 | 22.16 | 4.08 |
| 40 | 27.36 | 5.41 | 104.52 | 20.73 | 30.05 | 4.96 |
| 50 | 35.87 | 7.1 | 114.34 | 22.07 | 37.67 | 7.3 |
| 60 | 45.97 | 9.14 | 125.55 | 22.93 | 52.8 | 10.13 |
| 70 | 57.81 | 11.35 | 141.21 | 25.41 | 73.13 | 15.47 |
| 80 | 72.21 | 14.82 | 170.08 | 32.14 | 97.91 | 24.15 |
| 90 | 91.46 | 18.07 | 210.98 | 45.23 | 135.58 | 32.92 |
| 100 | 118.6 | 22.96 | 274.52 | 56.95 | 212.07 | 61.18 |
