# Supplementary material for: KCa3.1 K+ Channel Expression and Function in Human Bronchial Epithelial Cells
Source: PLoS One. 2015 Dec 21;10(12):e0145259. doi: 10.1371/journal.pone.0145259 (PMC4687003; doi:10.1371/journal.pone.0145259)
Supplement: S12 Table — Current values plotted against command potential (mV) values for currents recorded at baseline, and following the sequential addition of 1-EBIO and TRAM-34 from freshly brushed asthmatic HBECs. (PDF) [file pone.0145259.s015.pdf]

Command potential (mV)	Baseline		1-EBIO		TRAM-34	
-120	-121.11	67.15	-376	188.12	-118.23	51.06
-110	-109.33	60.55	-319.5	158.11	-105.65	44.08
-100	-90.92	49.99	-282.74	142.08	-99.53	43.59
-90	-84.05	50.12	-228.78	115.96	-87.28	42.16
-80	-76.68	49.39	-161.99	78.59	-73.89	38.26
-70	-63.56	40.67	-115.34	58.01	-60.53	38.62
-60	-54.93	33.46	-67.28	37.18	-48.03	35.37
-50	-43.84	29.07	-15.34	14.04	-34.38	28.97
-40	-30.56	18.72	31.6	6.95	-18.17	28.13
-30	-19.63	15.14	80.73	28.24	-5.78	25.97
-20	-14.02	13.06	131.21	49.77	12.67	26.05
-10	-7.41	8.37	181.68	72.35	25.35	25.95
0	2.32	1.92	233.74	94.55	41.91	26.56
10	11.72	2.62	285.83	113.97	55.89	28.41
20	21.93	5.87	334.43	139.03	68.3	31.48
30	29.44	13.22	382.2	156.9	84.57	34.34
40	40.36	16.16	426.45	181.8	96.21	41.66
50	50.88	23.95	456.73	202.14	112.55	44.35
60	61.01	28.22	482.2	219.11	129.98	51.15
70	67.46	33.56	505.94	238.91	143.17	58.95
80	81.36	40.38	520.83	261.85	153.52	64.2
90	97.51	48.97	537.72	286.01	172.83	73.69
100	113.81	58.66	554.85	303.16	195.78	82.85
