# Supplementary material for: KCa3.1 K+ Channel Expression and Function in Human Bronchial Epithelial Cells
Source: PLoS One. 2015 Dec 21;10(12):e0145259. doi: 10.1371/journal.pone.0145259 (PMC4687003; doi:10.1371/journal.pone.0145259)
Supplement: S13 Table — Current values plotted against command potential (mV) values for currents recorded at baseline, and following the sequential addition of 1-EBIO and TRAM-34 from freshly brushed healthy HBECs. (PDF) [file pone.0145259.s016.pdf]

Command potential (mV)	Baseline		1-EBIO		TRAM-34	
-120	-100.06	29.38	-196.13	48.94	-106.4	15.69
-110	-81.58	23.48	-155.35	35.53	-90.05	13.36
-100	-63.88	18.12	-126.51	28.92	-79.61	11.06
-90	-50.72	10.64	-93.04	25	-64.17	8.2
-80	-36.76	6.78	-56.62	22.01	-55.91	6.5
-70	-29.3	6.26	-22.83	26.44	-45.47	4.79
-60	-22.42	5.07	15.3	32.88	-34.77	3.16
-50	-14.89	5.72	50.52	41.56	-27.53	3.4
-40	-6.71	4.82	88.85	52.82	-20.83	5.74
-30	-1.49	4.92	119.47	63.45	-6.61	4.62
-20	7.67	6.63	152.79	72.78	2.46	4.75
-10	13.14	8.09	187.26	81.89	13.18	5.51
0	22.93	11.38	213.79	97.68	28.32	4.58
10	32.76	12.91	248.49	101.25	43.01	2.78
20	47.34	18.52	277.28	108.95	54.48	1.64
30	64.45	26.14	331.26	121.05	77.15	16.06
40	92.69	38.36	353.9	125.85	95.38	19.08
50	143.07	62.85	406.29	130.41	115.6	27.1
60	187.13	94.02	416.02	152.65	130	28.66
70	217.43	98.06	462.79	159.5	136.45	26.94
80	217.65	79.14	500.35	175	151.71	28.23
90	191.85	53.48	515.34	181.76	157.71	19.9
100	186.63	46.99	544.96	211.26	166.08	14.76
