# Supplementary material for: KCa3.1 K+ Channel Expression and Function in Human Bronchial Epithelial Cells
Source: PLoS One. 2015 Dec 21;10(12):e0145259. doi: 10.1371/journal.pone.0145259 (PMC4687003; doi:10.1371/journal.pone.0145259)
Supplement: S14 Table — Current values plotted against command potential (mV) values for currents recorded at baseline, and following the sequential addition of 1-EBIO and TRAM-34 from H292 cells. (PDF) [file pone.0145259.s017.pdf]

Command potential (mV)	Baseline		1-EBIO		TRAM-34	
-120	-68.85	9.74	-106.28	15.57	-116.61	59.88
-110	-54.27	9.23	-81.13	11.52	-107.63	56.23
-100	-43.13	6.29	-57.22	8.71	-93.36	50.81
-90	-33.47	4.68	-32.3	5.87	-79.68	42.69
-80	-27.09	3.38	-2.29	4.97	-72.18	40.13
-70	-21.21	3.78	32.14	9.02	-61.37	35.07
-60	-14.52	3.74	66.14	14.14	-49.26	28.33
-50	-11.52	2.24	103.1	20.06	-42.63	25.36
-40	-6.95	2.2	144.47	27.04	-31.49	19.5
-30	-3.77	2.49	188.47	33.74	-18.03	15.03
-20	1.8	1.59	232.39	41.86	-7.91	8.49
-10	7.5	2.41	279.74	49.59	1.9	3.94
0	10.73	4.52	332.28	59.18	13.68	3.2
10	17.93	3.46	380.09	67.71	25.63	7.64
20	20.8	5.51	435.79	78.63	36.06	13.12
30	27.56	5.77	482.79	87.31	47.43	16.96
40	29.8	7.74	526.81	95.73	56.4	20.12
50	35.83	7.67	566.3	108.28	68.69	24.04
60	39.52	8.07	599.13	116.27	81.86	27.35
70	50.28	10.05	631.81	127.65	93.33	32.48
80	57.22	13.57	660.93	135.04	109.94	36.25
90	72.5	17.41	687.02	140.32	127.13	43.24
100	83.01	20.27	694.3	142.71	138.73	51.35
