# Supplementary material for: KCa3.1 K+ Channel Expression and Function in Human Bronchial Epithelial Cells
Source: PLoS One. 2015 Dec 21;10(12):e0145259. doi: 10.1371/journal.pone.0145259 (PMC4687003; doi:10.1371/journal.pone.0145259)
Supplement: S15 Table — Current values plotted against command potential (mV) values for currents recorded at baseline, and following the sequential addition of 1-EBIO and TRAM-34 from BEAS-2B cells. (PDF) [file pone.0145259.s018.pdf]

Command potential (mV)	Baseline		1-EBIO		TRAM-34	
-120	-20.14	2.63	-194.97	49.15	-72.81	34.36
-110	-17.49	3.11	-163.46	42.85	-66.63	32.66
-100	-15.3	3.4	-120.55	34.24	-53.68	22.47
-90	-13.03	2.47	-72.39	26.2	-44.27	19.94
-80	-10.23	1.88	-24.87	21.88	-32.75	12.4
-70	-9.79	2.48	28.68	19.93	-27.51	10.81
-60	-7.56	2.13	85.92	23.36	-21.61	7.47
-50	-3.72	1.84	144.44	29.72	-13.83	7.07
-40	-2.83	2.1	212.67	38.04	-9.31	5.57
-30	-1.03	2.16	279.02	50.62	-2.98	1.98
-20	3.07	1.7	347.92	59.43	4.14	1.78
-10	4	2.4	422.88	69.85	12.87	4.34
0	5.4	1.91	484.08	83.12	17.35	6.34
10	8.08	2	560.83	93.92	26.57	9.67
20	10.49	1.92	635.03	105.34	33.15	12.08
30	13.62	2.94	710.5	119.75	39.88	14.56
40	17.14	2.88	765.3	131.03	47.39	15.29
50	22.92	2.97	847.91	136.15	55.71	15.94
60	24.87	4.86	911.29	140.86	59.26	20.14
70	34.41	7.56	945.16	153.85	67.77	20.62
80	41.08	7.46	998.38	157	73.98	19.62
90	49	11.53	1005.89	178.77	79.78	20.4
100	67.29	20	928.57	211.01	98.82	20.6
