# Supplementary material for: KCa3.1 K+ Channel Expression and Function in Human Bronchial Epithelial Cells
Source: PLoS One. 2015 Dec 21;10(12):e0145259. doi: 10.1371/journal.pone.0145259 (PMC4687003; doi:10.1371/journal.pone.0145259)
Supplement: S16 Table — ΔCT scores expressed as transcripts/106 18S mRNA of PCR reactions with MUC5AC TaqMan probes. (PDF) [file pone.0145259.s019.pdf]

PBS/BSA 6h	10ng/ml rh-AR 6h	100ng/ml rh-AR 6h
1	33.59	21.11
1	4.25	10.58
1	3.23	3.69

PBS/BSA 24h	10ng/ml rh-AR 24h
1	12.27
1	57.15
1	82.71
1	31.05
1	5.62
1	6.19
1	5.8
1	52.59
1	33.13

PBS/BSA 24h	100ng/ml rh-AR 24h
1	64.74
1	118.6
1	93.7
