# Supplementary material for: KCa3.1 K+ Channel Expression and Function in Human Bronchial Epithelial Cells
Source: PLoS One. 2015 Dec 21;10(12):e0145259. doi: 10.1371/journal.pone.0145259 (PMC4687003; doi:10.1371/journal.pone.0145259)
Supplement: S17 Table — ΔCT scores expressed as transcripts/106 18S mRNA of PCR reactions with MUC5AC TaqMan probes. (PDF) [file pone.0145259.s020.pdf]

DMSO	rh-AR + DMSO	rh-AR + TRAM-34
1.01	22.26	13.71
1.09	3.3	3.51
1.33	2.43	2.23
0.58	3.89	2.76
0.45	17.31	25.16
0.48	39.58	37.36
