# Supplementary material for: KCa3.1 K+ Channel Expression and Function in Human Bronchial Epithelial Cells
Source: PLoS One. 2015 Dec 21;10(12):e0145259. doi: 10.1371/journal.pone.0145259 (PMC4687003; doi:10.1371/journal.pone.0145259)
Supplement: S18 Table — Absorbance values detected at 450 nm. (PDF) [file pone.0145259.s021.pdf]

PBS/BSA	10ng/ml rh-AR	100ng/ml rh-AR
0.778	0.971	1.403
0.569	0.586	0.738
0.621	0.628	0.939
