# Supplementary material for: KCa3.1 K+ Channel Expression and Function in Human Bronchial Epithelial Cells
Source: PLoS One. 2015 Dec 21;10(12):e0145259. doi: 10.1371/journal.pone.0145259 (PMC4687003; doi:10.1371/journal.pone.0145259)
Supplement: S19 Table — Absorbance values detected at 450 nm. (PDF) [file pone.0145259.s022.pdf]

Unstimulated	10ng/ml rh-AR	100ng/ml rh-AR
1.326	1.386	1.917
1.164	1.322	1.782
1.269	1.394	1.85
1.083	1.514	1.841
