# Supplementary material for: KCa3.1 K+ Channel Expression and Function in Human Bronchial Epithelial Cells
Source: PLoS One. 2015 Dec 21;10(12):e0145259. doi: 10.1371/journal.pone.0145259 (PMC4687003; doi:10.1371/journal.pone.0145259)
Supplement: S20 Table — Absorbance values detected at 450 nm. (PDF) [file pone.0145259.s023.pdf]

DMSO	rh-AR + DMSO	rh-AR + TRAM-34
0.988	1.244	1.104
0.688	0.943	0.875
0.716	0.875	0.951
0.628	0.854	0.877
0.67	0.966	0.932
0.692	0.907	0.91
