# Supplementary material for: KCa3.1 K+ Channel Expression and Function in Human Bronchial Epithelial Cells
Source: PLoS One. 2015 Dec 21;10(12):e0145259. doi: 10.1371/journal.pone.0145259 (PMC4687003; doi:10.1371/journal.pone.0145259)
Supplement: S21 Table — Absorbance values detected at 450 nm. (PDF) [file pone.0145259.s024.pdf]

DMSO	rh-AR + DMSO	rh-AR + TRAM34
1.062	1.497	1.5
0.965	1.402	1.446
0.942	1.189	1.017
