# Supplementary material for: KCa3.1 K+ Channel Expression and Function in Human Bronchial Epithelial Cells
Source: PLoS One. 2015 Dec 21;10(12):e0145259. doi: 10.1371/journal.pone.0145259 (PMC4687003; doi:10.1371/journal.pone.0145259)
Supplement: S22 Table — Ciliary beat frequency (Hz) of epithelial cells from healthy controls. (PDF) [file pone.0145259.s025.pdf]

	Control		DMSO		TRAM-34	
0	12.70185	0.809943				
30			11.84444	0.7414374	11.74815	1.006813
60			11.75556	0.9275503	12.4963	0.8511029
