# Supplementary material for: KCa3.1 K+ Channel Expression and Function in Human Bronchial Epithelial Cells
Source: PLoS One. 2015 Dec 21;10(12):e0145259. doi: 10.1371/journal.pone.0145259 (PMC4687003; doi:10.1371/journal.pone.0145259)
Supplement: S23 Table — Ciliary beat frequency (Hz) of epithelial cells from asthmatic donors. (PDF) [file pone.0145259.s026.pdf]

	Control		DMSO		TRAM-34	
0	10.3	0.8				
30			11.7	1.2	10.3	1.1
60			11.7	0.8	11.4	0.8
