# Supplementary material for: KCa3.1 K+ Channel Expression and Function in Human Bronchial Epithelial Cells
Source: PLoS One. 2015 Dec 21;10(12):e0145259. doi: 10.1371/journal.pone.0145259 (PMC4687003; doi:10.1371/journal.pone.0145259)
Supplement: S24 Table — Values of ratio of cell length:cell width of vimentin-stained BEAS-2B cells. (PDF) [file pone.0145259.s027.pdf]

PBS/BSA	TGF-beta	TGF-beta + DMSO	TGF-beta + TRAM34	TGF-beta + ICA	TGF-beta + TRAM-7
2.05	3.59	4.69	2.65	2.65	3.36
2.07	3.72	3.6	2.18	2.47	3.29
1.86	3.38	3.95	2.17	2.43	2.91
2.1	2.79	3.94	2.43		
1.97	2.97	3.91	2.74		
1.64	2.83	3.11	2.07		
