# Supplementary material for: KCa3.1 K+ Channel Expression and Function in Human Bronchial Epithelial Cells
Source: PLoS One. 2015 Dec 21;10(12):e0145259. doi: 10.1371/journal.pone.0145259 (PMC4687003; doi:10.1371/journal.pone.0145259)
Supplement: S25 Table — Grayscale values of E-cadherin-stained BEAS-2B cells. (PDF) [file pone.0145259.s028.pdf]

Unstimulated	TGF-□1	TGF-□1 + DMSO	TGF-□1 + TRAM-34	TGF-□1 + ICA-17043	TGF-□1 + TRAM-7
7.1	1.2	1.1	6	5.3	2.8
5.1	1.9	1.9	2.7	4.4	1.4
4.9	1.8	1.6	4	4.4	2.5
4.7	2.1	2.2	3.1	5.6	3.2
5.8	1.3	0.4	3	4.1	1
4.2	0.8	1.1	3.2	1.9	0.6
