# Supplementary material for: KCa3.1 K+ Channel Expression and Function in Human Bronchial Epithelial Cells
Source: PLoS One. 2015 Dec 21;10(12):e0145259. doi: 10.1371/journal.pone.0145259 (PMC4687003; doi:10.1371/journal.pone.0145259)
Supplement: S26 Table — Grayscale values of collagen-1-stained BEAS-2B cells. (PDF) [file pone.0145259.s029.pdf]

PBS/BSA	TGF□1	TGF□1 + DMSO	TGF□1 + TRAM-34	TGF□1 + ICA-17043	TGF□1 + TRAM-7
28.8	33.8	31.8	28.19	27.62	31.31
20.7	34.8	31	23	23.56	25.19
21.9	32.5	26.4	21.94	21.61	27.08
34.7	74.3	61.3	44.93	50.99	61.57
17.9	23.6	40	30.14	21.98	30.65
